# Identifying model error in metabolic flux analysis – a generalized least squares approach

**DOI:** 10.1186/s12918-016-0335-7

**Published:** 2016-09-13

**Authors:** Stanislav Sokolenko, Marco Quattrociocchi, Marc G. Aucoin

**Affiliations:** Department of Chemical Engineering, University of Waterloo, 200 University Avenue West, Waterloo ON, Canada

**Keywords:** Metabolic flux analysis (MFA), Generalized least squares (GLS), Measurement uncertainty, *t*-test

## Abstract

**Background:**

The estimation of intracellular flux through traditional metabolic flux analysis (MFA) using an overdetermined system of equations is a well established practice in metabolic engineering. Despite the continued evolution of the methodology since its introduction, there has been little focus on validation and identification of poor model fit outside of identifying “gross measurement error”. The growing complexity of metabolic models, which are increasingly generated from genome-level data, has necessitated robust validation that can directly assess model fit.

**Results:**

In this work, MFA calculation is framed as a generalized least squares (GLS) problem, highlighting the applicability of the common *t*-test for model validation. To differentiate between measurement and model error, we simulate ideal flux profiles directly from the model, perturb them with estimated measurement error, and compare their validation to real data. Application of this strategy to an established Chinese Hamster Ovary (CHO) cell model shows how fluxes validated by traditional means may be largely non-significant due to a lack of model fit. With further simulation, we explore how *t*-test significance relates to calculation error and show that fluxes found to be non-significant have 2-4 fold larger error (if measurement uncertainty is in the 5–10 % range).

**Conclusions:**

The proposed validation method goes beyond traditional detection of “gross measurement error” to identify lack of fit between model and data. Although the focus of this work is on *t*-test validation and traditional MFA, the presented framework is readily applicable to other regression analysis methods and MFA formulations.

**Electronic supplementary material:**

The online version of this article (doi:10.1186/s12918-016-0335-7) contains supplementary material, which is available to authorized users.

## Background

As the metabolic phenotype of the cell, the flow of material through intracellular reactions (or metabolic flux) represents the sum total of all underlying cellular processes. The accurate determination of metabolic flux is becoming increasingly important for assessing the impact of metabolic engineering or feeding strategies on cellular metabolism [1]. In lieu of in vivo observation, the inference of intracellular fluxes is commonly accomplished through metabolic flux analysis (MFA). At its most basic, MFA refers to the process of modeling intracellular flux via a stoichiometric balance of metabolic reaction and transport rates (assuming a “pseudo steady-state” in the form of negligible molecule accumulation) [2]. The original applications of the technique centered on using simple element balances as a means to correct unreliable measurements [3]. However, the increasing availability of data from multi-omic technologies has led to the development of metabolic flux models that extend far beyond these foundations.

The basis of MFA is the stoichiometry matrix. In the typical arrangement, rows represent balances on molecular species, with each column encoding the stoichiometry of a reaction (see [2] for details). As cellular reaction networks generally have more reactions than species, the resulting stoichiometry matrix is typically underdetermined. The estimation of a single flux profile requires that the number of unknown reaction rates be equal to or less than the number of molecular species, and this has traditionally been accomplished by observing as many extracellular transport rates as possible. However, the growing availability of genomic data has opened the door to developing models that may contain thousands of reactions, complicating the calculation of a unique flux profile.

A considerable amount of metabolic information can be gathered without calculating a unique flux profile through constraint-based reconstruction and analysis (COBRA) methods. The combination of mass balance constraints from stoichiometric relations as well as other factors such as enzyme capacity and reaction thermodynamics can be used to generate a feasible solution space for cellular metabolism. If a unique flux profile is required, one can be estimated by assuming an objective function such as cell growth maximization. However, it is also possible to study the solution space directly (for a detailed review, see [4]). The popularity of COBRA methods has resulted in the development of a large number of software packages that have considerably simplified analysis (see [5]). However, the complexity of genome-scale models remains an on-going challenge.

Despite the recent advances, the process of translating genomic information to cellular reactions is still under development. Even the well-studied genomes of *Escherichia coli* and *Saccharomyces cerevisiae* had approximately 20 % of their open reading frames (ORFs) uncharacterized as recently as 2010 [6] and the development of reaction networks requires a significant amount of curation [6–8]. Furthermore, the relation between the presence of a gene sequence and enzymatic activity is not always obvious [7]. A combined transcriptomic-metabolomic modeling study of *E. coli* has revealed the existence of redundant gene expression where no flux was observed [9]. Meanwhile, a study of lysine-producing *Corynebacterium glutamicum* metabolism suggested that while the expression of some genes appears tightly coupled to metabolic fluxes, others can remain practically constant despite considerable changes in metabolic flux [10]. The popular Chinese Hamster Ovary (CHO) cell line has an added problem of high genetic variability that may question the generality of a given model [11, 12]. Taken together, these issues add a considerable amount of uncertainty to modeling efforts, especially for less studied expression systems.

The addition of isotopically labelled substrate and the analysis of resulting metabolites through ^13^C-MFA can be a powerful means to gain better understanding of a metabolic system. But despite the ready availability of algorithms and software packages to assist with everything from identifying optimal labelling strategies to final analysis (as reviewed in [13, 14]), ^13^C-MFA is not always practical. Isotopic labelling is expensive, especially for large volume bioreactor cultivation, and can not be used to monitor ongoing production processes. Moreover, studying transient labelling patterns requires accurate intracellular metabolite quantification, which is not always straightforward [15], and increased computational resources [13].

As such, one approach to dealing with genome-scale model uncertainty and complexity has been to simplify the models to a level where they can be solved directly from measured extracellular transport rates [16, 17], continuing the use of traditional overdetermined MFA. The simplification can be aided by software such as CellNetAnalyzer that can deal with both underdetermined COBRA models and overdetermined MFA formulations [18, 19]. Recent developments have also led to an automation of the model simplification process [20]. Despite increasing model size, overdetermined MFA has continued to see use over the last 10 years [16, 21–27], especially for less commonly used cell lines that lack well curated genomic and transcriptomic data. However, the reduction of genome-levels models in this fashion is an inversion of the original MFA foundations. In contrast to the use of a simple, reductive model for the reconciliation of questionable data, it is the accuracy of the model that is becoming increasingly variable – making it necessary to rigorously assess the validity of model simplification.

A number of strategies are currently available for model validation. The stoichiometric matrix can be probed directly by checking its condition number [28] or by determining the sensitivity of calculated fluxes to measurement error [29]. The incorporation of measurement flux uncertainty allows the use of gross measurement error detection [30], which identifies whether deviations between observed and fit data are normally distributed through a *χ*^2^-test. While useful for identifying singular errors of large magnitude, this statistic does not asses the overall quality of fit – errors may be unreasonably large while remaining normally distributed. Despite the increasing consideration of confidence intervals around calculated fluxes in recent studies [31, 32], the question of whether a set of data fits a given metabolic model has thus far remained open.

In this work, we propose the use of a standard *t*-test as a natural extension of the least-squares calculation that underpins traditional MFA calculation. Applying MFA to a Chinese Hamster Ovary (CHO) cell culture, *t*-tests were used to determine whether each calculated flux could be deemed sufficiently distinct from zero. Once non-significant fluxes were identified, we explored whether the uncertainty in calculated fluxes could be explained by measurement uncertainty alone, or if a lack of model fit could be to blame. To do this, the solution space of the stoichiometric model was constrained by observed flux ranges and hypothetical flux profiles were generated directly from the model. The profiles were perturbed by measurement error and collected to establish a baseline of calculated flux significance given perfect model fit.

## Methods

### Theoretical principles^1^

The material balance on molecular species that forms the basis of MFA is typically expressed as

1 $$ S v = 0  $$

where *S* is the stoichiometric matrix and *v* is the vector of fluxes that correspond to reactions defined by columns of *S*. This formulation proceeds from a pseudo steady-state assumption that changes in metabolite pools (as a result of cell division or other processes) are much smaller than metabolite production and consumption fluxes and can therefore be ignored. The *Sv* matrix can be be separated into *S*_*c*_*v*_*c*_+*S*_*o*_*v*_*o*_, where *c* stands for calculated flux and *o* for observed flux.

2 $$\begin{array}{*{20}l} S_{c} v_{c} + S_{o} v_{o} &= 0 \end{array} $$

3 $$\begin{array}{*{20}l} -S_{o} v_{o} &= S_{c} v_{c} \end{array} $$

Since *v*_*o*_ is a vector of observed data, *S*_*o*_*v*_*o*_ can be calculated directly. The dimension of *S*_*c*_ depends on how many fluxes can be observed, i.e., the length of *v*_*o*_. *S*_*c*_ must have no more columns than rows to calculate a unique flux profile, although the observation of more fluxes (and the accompanying reduction in the number of *S*_*c*_ columns) is useful for error estimation^2^. Pooling cyclic or parallel pathways may be required in the initial formulation of *S* to ensure the required form of *S*_*c*_ is obtained.

Assuming that an overdetermined form of *S*_*c*_ can be formulated (with sufficient information to calculate *v*_*c*_), Eq. (3) is equivalent to linear regression and can be solved in a similar fashion.

**Table:** 

Linear regression	MFA
$y = X\beta + \varepsilon \phantom {\dot {i}\!}~~~~~~~~~~~~~~~~~~(4)$	$\phantom {\dot {i}\!}-S_{o} v_{o} = S_{c} v_{c} + \varepsilon ~~~~~~~~~~~(5)$
$\phantom {\dot {i}\!} \hat {\beta } \,=\, \left (X^{T} X \right)^{-1} X^{T} y~~~~~(6)$	$\phantom {\dot {i}\!}\hat {v}_{c} = -\left ({S_{c}^{T}} S_{c} \right)^{-1} {S_{c}^{T}} S_{o} v_{o}~~~(7) $

With this formulation, *ε* represents the deviation between observed and calculated fluxes that may be the result of either measurement error or lack of model fit. Equation (7) assumes *ε* is independently and identically distributed, which is unlikely to be the case. The variance-covariance matrix Cov(*ε*) can be expressed as a scalar *σ*^2^ multiplied by a matrix of relative covariance terms *V*, i.e., Cov(*ε*)=*σ*^2^*V*. If observed fluxes do not covary and have equal variance, then *V*=*I*, where *I* is the identity matrix. Otherwise, Eq. (5) needs to be rescaled by the matrix square root of *V*. Taking *V*=*PP*, the scaled form of Eq. (5) is:

8 $$ -P^{-1}S_{o} v_{o} = P^{-1}S_{c} v_{c} + P^{-1}\varepsilon  $$

where *P*^−1^*ε* now satisfies the assumptions of linear regression. Formally, this is equivalent to generalized least squares (GLS) regression; however, incorporating *P*^−1^ directly into each term allows the use of all ordinary least squares techniques. Letting *P*^−1^*S*_*o*_=*So*′, *P*^−1^*S*_*c*_=*Sc*′, and *P*^−1^*ε*=*ε*^′^:

9 $$\begin{array}{*{20}l} \hat{v}_{c} &= -\left(S_{c}^{\prime T} S'_{c} \right)^{-1} S_{c}^{\prime T} S'_{o} v_{o} \end{array} $$

The calculation of *P*^−1^ requires the estimation of Cov(*ε*) from the variance of observed fluxes. Calculating the covariance-variance matrix of both sides of Eq. (5):

10 $$\begin{array}{*{20}l} \text{Cov}\left(-S_{o} v_{o}\right) &= \text{Cov}\left(S_{c} v_{c} + \varepsilon\right) \end{array} $$

11 $$\begin{array}{*{20}l} \text{Cov}(\varepsilon) &= S_{o} \text{Cov}(v_{o}) {S_{o}^{T}}  \end{array} $$

Since Cov(*ε*)=*σ*^2^*V* for any value of *σ*, *σ* is set to 1 so that *V*=Cov(*ε*). In practice, Cov(*v*_*o*_) need only capture the relative magnitudes of observed flux variances as $\hat {\sigma }$ is estimated during regression. Balances around molecular species that do not include an observed flux *v*_*o*_ will have a row of zeros in Cov(*ε*), which prevents the calculation of a matrix inverse (required to get *P*^−1^). Although this mathematically equates to a variance of zero for those balances, a better interpretation is that there is an unknown variance around the “observation” of no net flux. The simplest solution is to add a small non-zero value to each diagonal entry of Cov(*ε*), representing the confidence of the calculated fluxes being fully balanced. If there is more uncertainty around some balances than others, this information could be encoded in the magnitude of the added variance. *P* can then be calculated via a matrix square root of estimated Cov(*ε*). Since a variance (covariance) matrix is positive semi-definite, *P* is known to be unique.

Whereas calculated fluxes $\hat {v}_{c}$ are commonly estimated using a very similar “weighted” least squares approach, the use of validation methods that are part of the regression framework have yet to be explored. The common *χ*^2^ test can still be used to detect gross measurement errors in estimated residuals ($\hat {\varepsilon }$); however, the validation of a regression model also requires the use of *t*-tests to ensure the significance of calculated fluxes. Confidence and prediction intervals are also highly relevant to MFA. Estimated fluxes require a confidence interval to report the uncertainty of calculation, while a prediction interval around a predicted balance can be used to judge the validity of that balance being closed. The calculation of a *t*-statistic follows from normal regression:

**Table:** 

Linear regression	MFA
$\phantom {\dot {i}\!} t_{\hat {\beta }_{i}} = \frac {\hat {\beta }_{i}}{\text {se}(\hat {\beta }_{i})}~~~~(12) $	$\phantom {\dot {i}\!} \;\; t_{\hat {v}_{c,i}} = \frac {\hat {v}_{c,i}}{\text {se}(\hat {v}_{c,i})}~~~~(13) $

Thus:

14 $$ t_{\hat{v}_{c,i}} = \frac{\left(-\left(S_{c}^{\prime T} S'_{c} \right)^{-1} S_{c}^{\prime T} S'_{o} v_{o} \right)_{i}}{\hat{\sigma} \sqrt{\left(S_{c}^{\prime T} S_{c}'\right)^{-1}_{i,i}}}  $$

The estimated standard deviation of *ε* (or $\hat {\sigma }$) is calculated as follows:

15 $$ \hat{\sigma}^{2} = \frac{\sum \left(\hat{\varepsilon}_{i}'\right)^{2}}{n_{b} - n_{c} - 1}  $$

where:

16 $$ \hat{\varepsilon}' = -S'_{o} v_{o} + S'_{c} \left(S_{c}^{\prime T} S'_{c} \right)^{-1} S_{c}^{\prime T} S'_{o} v_{o}  $$

and *n*_*b*_ is the number of balances (rows of $S^{\prime }_{c}$) while *n*_*c*_ is the number of fluxes to be calculated (columns of $S^{\prime }_{c}$). If the model is correct and Cov(*ε*) was correctly estimated, $\hat {\sigma }^{2}$ should be approximately equal to 1. Once the *t*-value is calculated, a flux can be judged statistically significant if $|t_{\hat {v}_{c,i}}| \ge t_{\alpha /2, n_{b} - n_{c} - 1}$ where *α* is the significance level.

The identification of non-significant flux may be interpreted in two ways. The measurement error around observed fluxes may be too high to allow robust flux calculation. In that case, non-significant fluxes should be treated as having a flux of zero and excluded from the model or further analysis. Alternatively, non-significance may be the result of excess variability from a lack of fit between the model and observed data, requiring model correction. To distinguish between these cases, it is necessary to separate model error from measurement uncertainty. One way to accomplish this is to reduce measurement uncertainty through added replication; however, the required effort can make this approach practically infeasible. Another solution is to simulate a set of feasible fluxes directly from the stoichiometric model (and therefore free of model error) for comparison to the observed data.

The simulation of feasible fluxes can be simplified by eliminating flux equality constraints expressed by the stoichiometry matrix. Essentially, only *n*_*c*_−*n*_*b*_ fluxes have to specified in order to generate all the other values. More formally, the relationships between the fluxes can be succinctly summarized through the nullspace (or kernel) of *S*, which describes all flux balance conservations in the model. This makes it possible to calculate all fluxes from a smaller set of variables referred to as the basis. Unlike fluxes, which must satisfy constraints imposed by *Sv*=0, the basis can take any arbitrary value to generate fluxes that satisfy all required constraints. Expressed mathematically,

17 $$\begin{array}{*{20}l} \text{Null}(S) &= K \end{array} $$

18 $$\begin{array}{*{20}l} Kb &= v \end{array} $$

where *b* is a basis vector of any value with the same number of rows as columns of *K*. While all values of *b* satisfy *Sv*=0, it is still necessary to constrain fluxes to a set of realistic values representative of a cell cultivation. The space of all feasible fluxes *v* can be constrained by defining upper and lower bounds on each observed flux:

19 $$ \begin{array}{ll} & v = Kb \\ \text{subject to}& K_{i}b \leq v_{i} + a \cdot \text{sd}(v_{i}) \\ & K_{i}b \geq v_{i} - a \cdot \text{sd}(v_{i}) \end{array}   $$

where *v*_*i*_ is an observed flux, *K*_*i*_ is the corresponding row of *K*, and *a* is a scaling constant that can be set to *t*_*α*/2,*df*_ to specify a confidence interval around *v*_*i*_. As the basis solution space is only constrained by inequalities, it is readily amenable to stochastic sampling. All values of *v* that satisfy Eq. (19) represent feasible fluxes that would perfectly satisfy the stoichiometric model while remaining within measurement uncertainty of real observations. If the resulting space is infeasible, then the observed data does not fit the specified model. Otherwise, a random sample of feasible fluxes can be taken for comparison to observed results. If the addition of measurement error to simulated fluxes results in less uncertainty than from observed results, then model error is to blame.

### Cell culture

CHO-BRI cells were grown in a 3 L bioreactor (Applikon Biotechnology Inc., Foster City, CA) in serum-free BioGro-CHO media (BioGro Technologies Inc., Winnipeg, Canada) with an in-house amino acid supplement (manuscript submitted). The culture was seeded at 0.3·10^6^ cells/ml with a working volume of 2 L. Temperature, pH, dissolved oxygen, and agitation speed were held at 37 °C, 7.4, 50 %, and 120 RPM respectively. Samples were taken three times a day for offline analysis. Cell density was determined using a Coulter Counter Z2 (Beckman Coulter, Miami, FL) calibrated to results from trypan blue exclusion analysis. Aliquots were centrifuged, with the supernatant collected and stored at -80 °C until Nuclear Magnetic Resonance (NMR) analysis. Dry cell mass was calculated by vacuum filtering 15 mL of cell culture through a type A/D glass filter (Pall Corporation, Port Washington, NY) and weighing the filter after drying it for 24 hours at 50 °C.

### Metabolite quantification

NMR spectra acquisition, metabolite quantification, and internal standard correction are described in [33]. In brief, samples were scanned on a Bruker Avance 600 MHz spectrometer using the first increment of a 1D-NOESY pulse sequence with metabolite quantification carried out using Chenomx NMR Suite 8.1 (Chenomx Inc., Edmonton, Canada). GlutaMAX™ was added manually to the software library using the Chenomx NMR Suite’s ‘compound builder’ tool. All compounds were profiled in triplicate. Ammonia measurements were taken using an Orion Star™Plus ISE Meter (Thermo Fisher Scientific, Waltham, MA).

### MFA model

A CHO cell MFA model was taken from [34]. New transport fluxes were added for acetate, formate, pyruvate, citrate, malate, pyroglutamate, and GlutaMAX™ (the fluxes of which could all be observed via NMR). The transport of GlutaMAX™ was grouped together with the conversion of the dipeptide into glutamine and alanine. The transport of cystine was grouped together with the reduction of cystine into cysteine. A new reaction was added for the conversion of glutamate into pyroglutamate [35] (via a number of possible enzymatic and non-enzymatic reactions). New reactions were also added for acetyl-CoA hydrolase and formate-tetrahydrofolate ligase to explain acetate and formate production. Along with a Systems Biology Markup Language (SBML) representation of the model, a full list of reactions and an outline of metabolite flow are provided as Additional files 1, 2 and 3. As in the original formulation, a number of unbalanced species were removed from the model before analysis, including O_2_, CO_2_, ATP, NADH, NADPH, and FADH (NADH and NADPH were later reintroduced in a modified form of the model).

### Flux estimation

Metabolite and cell concentration timecourse data was fit by a regression spline with 4 cubic basis functions (provided by the gam function [36] in the R programming language [37]). Measurement error was estimated by calculating the variance of observation deviation from the fit. 1000 predicted concentration timecourses were simulated for each trend by adding normally distributed error corresponding to the sum of regression and measurement variance. A new regression split fit was calculated for each of the simulated timecourses. Metabolite transport fluxes were calculated by dividing the derivative of the metabolite concentration fit by cell concentration ($v_{o} = \frac {1}{X}\frac {\mathrm {d}C_{o}}{\mathrm {d}t}$). The mean and variance of the simulated fluxes at each time-point were used for all MFA analysis. Biomass fluxes were calculated as in [34], with the exception that dry cell mass measured to be 0.24 mg/ 10^6^ cells. A single mid-exponential time-point of 66 hours was chosen for MFA analysis to fulfill pseudo steady-state conditions.

### Implementation

All MFA calculations, validation, and sampling were carried out using the omfapy Python package, developed in-house. The package as well as analysis code is available on github (https://github.com/ssokolen/omfapy). Basic functionality was based on theoretical principles presented in [2]. Sampling of a feasible flux space was implemented using the random direction algorithm [38] as well as the mirror algorithm presented in [39]. Although slower, the mirror algorithm was able to generate more even coverage of the sampling space.

## Results

### Identification of model error

**Fig. 1:**
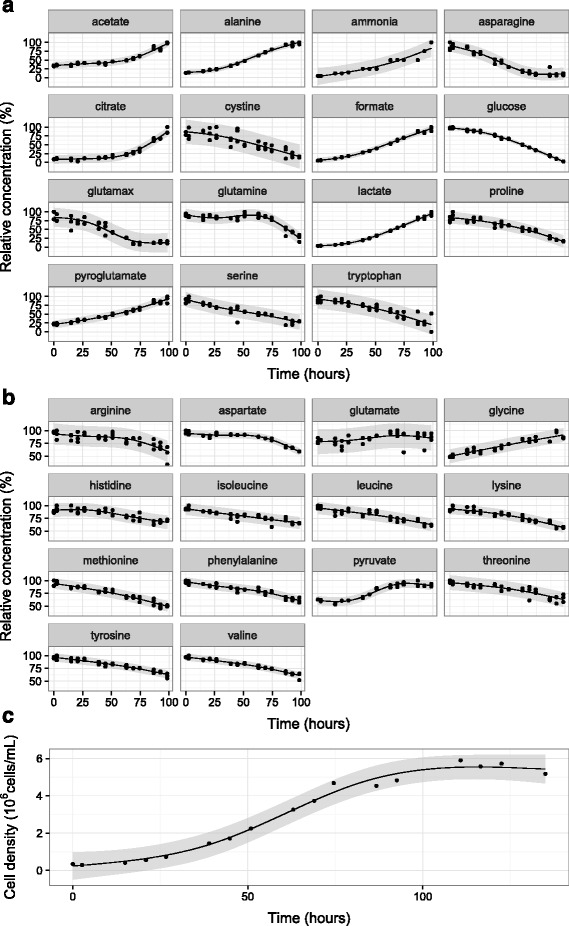
Observed time-course trends. The presented data depicts all metabolic trends from a CHO cell cultivation carried out in a batch reactor (see Cell culture section of Methods for more detailed information). A single timepoint of 66 hours was chosen for MFA analysis, corresponding to the midpoint of the exponential phase (where the cells are likely to grow under pseudo steady state conditions). Panels depict **a** metabolites that changed by more than 50 % of their maximum concentration, **b** those that changed by less than 50 %, and **c** cell density. All metabolite concentrations are expressed as fractions of their maximum value. Curves were calculated from cubic regression spline fits constrained to 4 basis functions. Grey area designates 99 % prediction interval used for sampling

Observed uptake fluxes and their corresponding coefficients of variation 66 hours post inoculation are shown in Table 1, with overall metabolite concentration profiles and cell density in Fig. 1. As usual for CHO cells, the metabolic profile was dominated by large fluxes of glucose and lactate. Considerable fluxes of alanine, GlutaMAX^TM^, ammonia, and glutamine were also observed. The median coefficient of variation was found to be 9.3 %. Although this was similar to previously reported estimates for concentration quantification via NMR [40], incorporating the uncertainty of derivative calculation resulted in a somewhat larger probability of high variance values. As in [40], the singularly high variability of glutamate flux was primarily due to its low concentration and heavy spectral convolution.

**Table 1:** Observed uptake fluxes and coefficients of variation (standard deviation of flux divided by flux) 66 hours post inoculation

	Flux $\left (\frac {\text {nmol}}{10^{6} \text {cells} \cdot \mathrm {h}}\right)$	CV (%)
Acetate	-1.03	5.08
Alanine	-33.95	3.32
Ammonia	-17.65	23.10
Arginine	2.52	16.33
Asparagine	2.21	7.64
Aspartate	2.14	7.09
Carbohydrates	-2.13	12.25
Citrate	-1.56	7.14
Cystine	0.33	19.04
DNA	-0.31	13.15
Formate	-7.52	2.06
Glucose	161.87	2.89
Glutamate	-0.17	213.18
Glutamax	17.98	10.69
Glutamine	7.35	12.48
Glycine	-2.25	8.79
Histidine	1.02	14.92
Isoleucine	1.52	8.13
Lactate	-283.53	3.19
Leucine	2.66	9.47
Lipids	-1.36	14.86
Lysine	1.80	8.05
Malate	-0.40	13.78
Methionine	0.89	6.44
Phenylalanine	1.19	7.04
Proline	1.94	9.17
Protein	-32.69	13.11
Pyroglutamate	-3.86	3.86
Pyruvate	-2.62	5.74
RNA	-0.89	13.77
Serine	2.64	12.36
Succinate	-0.15	15.52
Threonine	1.70	11.45
Tryptophan	0.34	17.40
Tyrosine	1.11	6.57
Valine	2.24	5.26

The incorporation of the observed fluxes into the MFA model showed no issues using typical metrics. The condition number of the reduced stoichiometry matrix was considerably below 1000 and the *χ*^2^*p*-value was 0.93, indicating little evidence of gross measurement error. However, *t*-test analysis on the calculated fluxes using the GLS framework revealed that only 15 of 47 fluxes were statistically significant (at the standard 5 % significance level). The statistically significant fluxes were primarily those that related to glycolysis – offering only a shallow look at cellular metabolism. All of the TCA and many of the amino acid degradation fluxes were deemed non-significant. To determine whether measurement variability or model error was to blame, 100 flux profiles were sampled from the stoichiometric matrix bounded by 99 % confidence intervals on the measured fluxes (fluxes generated directly from the model in this way will be referred to as “balanced”). Ninety-nine percent intervals were chosen to include practically all possible flux values. The sampled fluxes had good coverage of the constraint space, suggesting that the model was flexible enough to fit fluxes similar to those observed. Each balanced flux profile was then perturbed 100 times using normally distributed noise generated from observed flux standard deviations. The result was 10 000 sets of fluxes subject to observed measurement error but no model error.

**Fig. 2:**
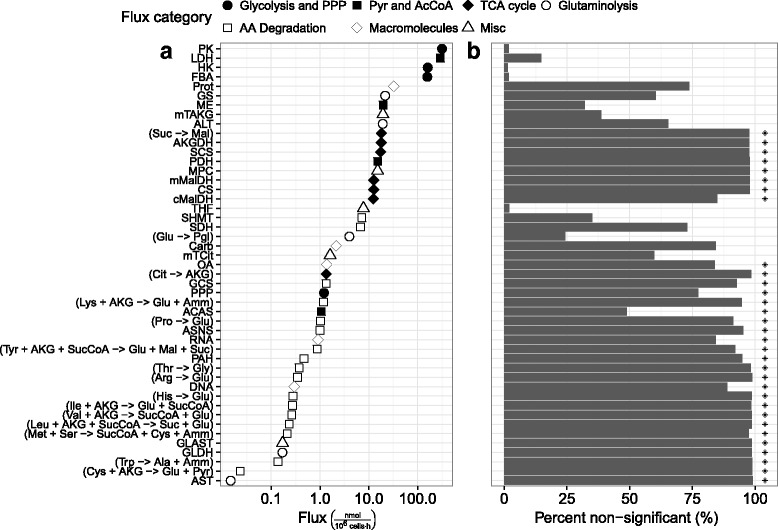
Comparison of flux rejection between observed and simulated data. Panels depict **a** calculated flux magnitude and **b** the percent of simulations in which the calculated fluxes were found to be non-significant (with asterisks indicating fluxes calculated to be non-significant using observed data). Simulated data was drawn from the stoichiometric model described in the Methods section, constrained by 99 % confidence intervals on fluxes observed at 66 hours post inoculation. 100 balanced flux profiles were generated with 100 random generated sets of measurement error applied to each. See Additional file 2 for reaction definitions

Figure 2 compares the percentage of simulated (balanced) fluxes found to be non-significant to the results from observed data. The simulation revealed that approximately half of the calculated fluxes (and all TCA fluxes) are entirely non-significant even when there is no model error (Fig. 2b). Many of the other fluxes were only significant for 50 % of the simulations or fewer. The lack of significance showed that the model was incapable of providing high confidence results for the collected data. Along with the overall low significance, evidence of model error could also be observed. Focusing on approximately 20 of the lowest magnitude fluxes, all were deemed to be non-significant based on the observed data. Comparing the simulated data, the same fluxes were rejected as non-significant 50–95 % of the time. Taken together, the probability of all the low magnitude fluxes being observed as non-significant is extremely low, giving strong indication of poor fit beyond the effect of measurement error alone, i.e., as a result of model error. Although model correction is outside the scope of this work, the proposed methodology was successful in identifying a considerable degree of uncertainty overlooked by commonly used validation methods.

### Effect of measurement noise

**Fig. 3:**
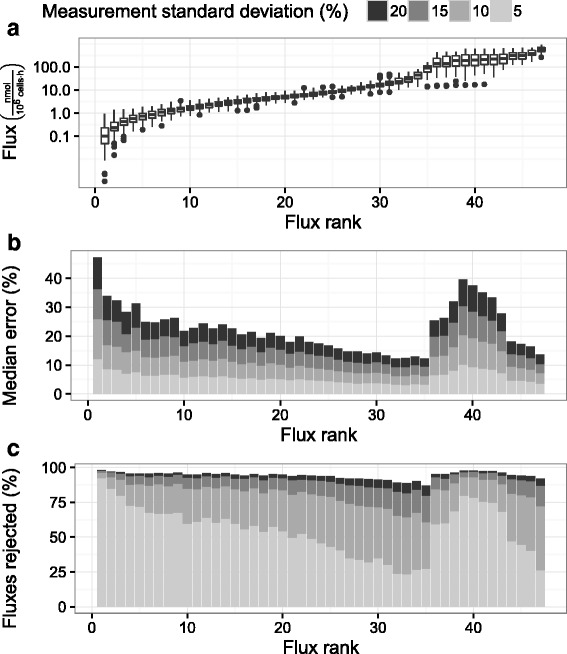
Comparison of fluxes simulated with different measurement errors. Panels depict **a** flux magnitude, **b** median error, and **c** percent non-significance. Simulated data was drawn from the stoichiometric model described in the Methods section, constrained by 99 % confidence intervals on fluxes observed between 18 and 80 hours post inoculation. 100 balanced flux profiles were generated with 100 random generated sets of measurement error applied to each. Each balanced flux profile was ordered according to increasing absolute flux magnitude to generate an associated rank from 1 to 45

An extended simulation was carried out to determine whether the lack of statistical significance was due to measurement variability. The flux constraints were extended beyond 66 hours post inoculation to consider the broader applicability of the model. 99 % confidence intervals were generated for all fluxes 18-80 hours post inoculation with the minimum and maximum values for each flux used to bound the flux solution space. 100 balanced flux profiles were generated with 100 sets of measurement error drawn from a normal distribution using 5, 10, 15, and 20 % coefficients of variation for each flux. The 45 calculated fluxes spanned more than 3 logarithms of values from approximately 0.1 $\frac {\text {nmol}}{10^{6} \text {cells} \cdot \mathrm {h}}$ to 400 $\frac {\text {nmol}}{10^{6} \text {cells} \cdot \mathrm {h}}$ (Fig. 3a). Fluxes had variable magnitudes across the simulations, so all analysis was performed as a function of flux rank, where a rank of 1 indicates the smallest magnitude flux in a given flux profile.

All the simulated flux profiles were subject to a *χ*^2^ test, with only 5 % of the simulations rejected (equal to the false positive rate). The remainder of the fluxes are shown in Fig. 3. As the simulated fluxes included both observed and calculated values, a percent error could be calculated for each calculated flux. Despite passing the *χ*^2^ test, most fluxes were characterized by median errors of 10–20 % (Fig. 3b), increasing with measurement variability. It should be noted that the median is a relatively conservative statistic. By definition, half of the calculated fluxes featured much greater errors than the reported values. The pronounced jump in error for flux ranks of 36 to 44 was traced to the TCA fluxes, which had high error despite large flux magnitudes. Similar to median error, the percentage of fluxes identified as non-significant increased with measurement variability (Fig. 3c). However, even measurements with 5 % coefficient of variation resulted in rejection rates of 50 % or more across practically all fluxes. The TCA fluxes in particular (ranks 36 to 44) were rejected as non-significant 75 % of the time or more (at all levels of measurement variability). The high level of flux rejection at low levels of measurement variability suggested the uncertainty in MFA calculation using observed data was primarily due to model structure rather than the uncertainty of observed data. Despite passing traditional validation tests, the simulation of stoichiometrically balanced fluxes revealed that the model is incapable of explaining observed metabolic profiles with an acceptable degree of confidence.

### Effect of model structure

**Fig. 4:**
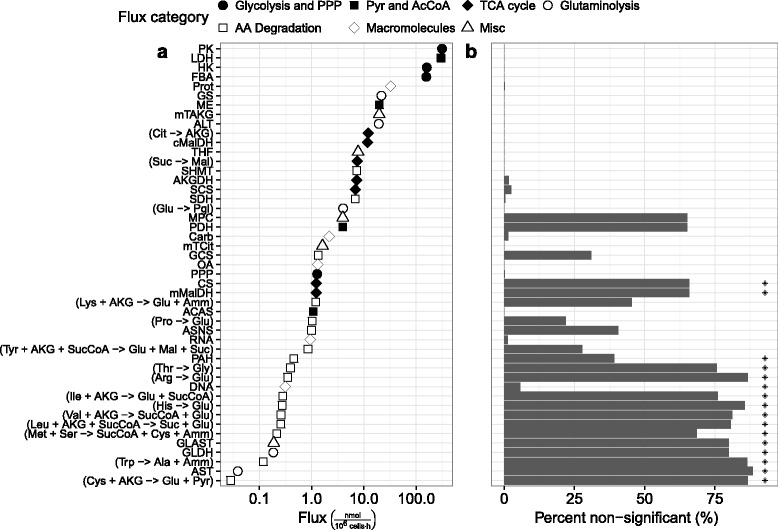
Comparison of flux rejection between observed and simulated data following model modification. Panels depict **a** calculated flux magnitude and **b** the percent of simulations in which the calculated fluxes were found to be non-significant (with asterisks indicating fluxes calculated to be non-significant using observed data). Simulated data was drawn from a modification of the stoichiometric model described in the Methods section (with balances on NADH and NADPH), constrained by 99 % confidence intervals on fluxes observed at 66 hours post inoculation. 100 balanced flux profiles were generated with 100 random generated sets of measurement error applied to each. See Additional file 2 for reaction definitions

To test the influence of model structure on the significance of calculated fluxes, we simulated the effect of a broken electron transport chain – allowing a closed balanced on NADH and NADPH. Essentially, NADH and NADPH were reintroduced into the model and assumed to be balanced by the defined stoichiometric relations. Although arbitrary, this assumption is consistent with largely anaerobic metabolism of CHO cells (termed the “Warburg Effect”) and allowed the addition of balances around intermediate compounds participating in many reactions. Incorporating the modified model into analysis of the observed fluxes at 66 hours post inoculation revealed no sign of gross measurement error (*χ*^2^*p*-value of 0.91) and decreased the number of non-significant fluxes from 32 (of 47) to 16. As before, 10 000 sets of fluxes were simulated from 99 % confidence intervals around the observed measurement fluxes, subject to observed measurement error (Fig. 4). In comparison to Figs. 2b, Fig. 4b reveals a considerable increase in significance across a large number of fluxes, consistent with the idea that model structure plays an important role in uncertainty around calculated fluxes. The impact was particularly drastic for TCA fluxes, most of which changed from entirely non-significant to significant. Despite the improvement in model fit, some model error could also be observed – too many of the low magnitude fluxes calculated from observed data were found to be non-significant when compared to the simulated results.

**Fig. 5:**
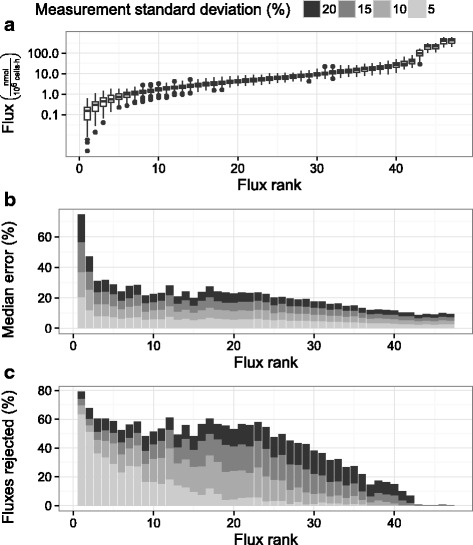
Comparison of fluxes simulated with different measurement errors following model modification. Panels depict **a** flux magnitude, **b** median error, and **c** percent non-significance of fluxes simulated with different measurement errors. Simulated data was drawn from a modification of the stoichiometric model described in the Methods section (with balances on NADH and NADPH), constrained by 99 % confidence intervals on fluxes observed between 18 and 80 hours post inoculation. 100 balanced flux profiles were generated with 100 random generated sets of measurement error applied to each. Each balanced flux profile was ordered according to increasing absolute flux magnitude to generate an associated rank from 1 to 45

**Fig. 6:**
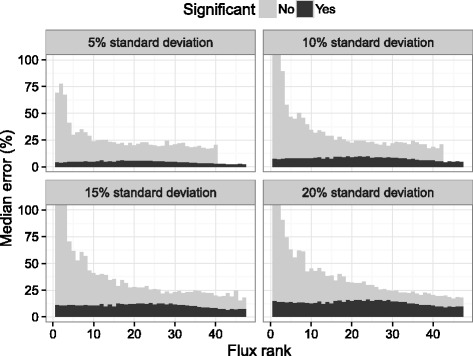
Comparison of median error of significant and non-significant fluxes (determined by *t*-test with *α*=0.05) simulated with different measurement errors. Simulated data was drawn from a modification of the stoichiometric model described in the Methods section (with balances on NADH and NADPH), constrained by 99 % confidence intervals on fluxes observed between 18 and 80 hours post inoculation. 100 balanced flux profiles were generated with 100 random generated sets of measurement error applied to each. Each balanced flux profile was ordered according to increasing absolute flux magnitude to generate an associated rank from 1 to 45

The modified model was also tested with an extended simulation (Fig. 5). As with the original model, 99 % confidence intervals were generated for all fluxes 18–80 hours post inoculation with the minimum and maximum values for each flux used to bound the flux solution space. The most pronounced impact of the modification was on the rate of flux rejection (Fig. 5c). At 5 % measurement variability, approximately two thirds of the fluxes were always significant. The remaining third of the lowest magnitude fluxes were significant at least 50 % of the time. In comparison, none of the fluxes calculated with the original model were significant for more than 75 % of the simulations. To get a better idea of how the *t*-test metric related to flux inaccuracy, median errors were separated for significant and non-significant fluxes. At 5 % coefficient of variation, fluxes deemed statistically significant had a constant median error of less than 5 % (with relation to flux rank), while non-significant fluxes had considerably higher errors (Fig. 6). Increasing coefficients of variation resulted in dramatic increases in overall rates of flux rejection (Fig. 5c). However, the median error of statistically significant fluxes also increased, diminishing the ability of the *t*-test metric to identify inaccuracy in higher magnitude fluxes (Fig. 6). In comparison, the typical *χ*^2^ test retained a 5 % rejection rate for all measurement errors (equal to the false positive rate).

## Discussion

Taken together, the results of the simulations suggest that both measurement uncertainty and model structure have an impact on MFA results that are not assessed by typical validation methods. The structure of the model may lead to a considerable amount of uncertainty around calculated fluxes despite a high level of measurement precision. Mathematically, this impact can be seen in the $\left (S_{c}^{\prime T}S_{c}^{\prime }\right)^{-1}$ term that stems from the variance of estimated regression parameters, i.e., $\text {Cov}(\hat {\beta })$. Less formally, it may be intuitive that a model featuring a balance on important intermediate metabolites such as NADH and NADPH would be able to estimate intracellular fluxes with a greater degree of confidence than a model without the extra information afforded by the balance. Naturally, the addition of isotopically labelled substrates can add a much greater degree of certainty. Indeed, an important application of the proposed testing and simulation framework is to provide a rigorous assessment of when extra information from sources such as labelled substrate would be essential for accurate flux calculation.

The proposed framework integrates a number of validation steps. While the *t*-test offers a straightforward post-regression significance test, combining the *t*-test with balanced flux simulation provides a convenient assessment of practical model identifiability [41, 42]. In addition, comparing the results from simulated and observed values can identify a lack of fit between model and measured data. Model fit is particularly important in the context of overdetermined MFA due to the large degree of simplification involved in model generation. Our findings suggest that the results of such simplification may be poor identifiability and lack of fit. These issues are rarely considered outside of “gross measurement error” detection. The combination of *t*-test validation and balanced flux simulation offers a simple and practical approach that avoids the assumption of model validity in the determination of significance. Although this validation strategy was developed for the analysis of simplified metabolic models, it should be equally useful at larger scales provided that enough observations are available.

It is important to note that the GLS framework for validation is more robust to estimated measurement error than the standard *χ*^2^ test. GLS regression only requires an estimate of relative measurement variance and covariance in the form of *V*. Residual variance magnitude ($\hat {\sigma }^{2}$) is still estimated from the model. On the other hand, variance scaling in the *χ*^2^ test allows for large measurement variance to reduce the *χ*^2^ statistic. Effectively, high variability leads to a lower confidence that deviations are not normally distributed. Given that variance does not factor into any other aspect of validation, assuming a large variance can serve as a way to avoid dealing with lack of fit.

Following the case study presented in this work, we recommend the following validation procedure. Before any experiments are carried out (but after a model of interest has been identified), construct reasonable limits around each observable flux from literature or other available data. Simulate flux profiles from the constrained flux space and perturb them with a range of measurement errors. If the flux space is infeasible, there is considerable disagreement between fluxes and the model that needs to be resolved. Otherwise, generate confidence intervals around the calculated fluxes and calculate the proportion of simulated fluxes that are non-significant. If many high magnitude fluxes are found to be non-significant in the majority of simulations (regardless of measurement error), then the model may have structural issues that need to be resolved. Alternatively, extra flux information may be required. If the model is sound, then experiments can be carried out and collected data analyzed via MFA. Apply the model and generate confidence intervals around calculated fluxes. Construct limits in close vicinity of observed values, simulate flux profiles, and perturb them with estimated measurement error. If the confidence intervals of simulated fluxes are considerably smaller than those of observed fluxes, then the model may have errors resulting in a lack of fit.

## Conclusion

The interpretation of MFA through the GLS framework underscores the need for robust validation methods. The mathematical equivalence of MFA and regression suggests that the failure to follow good practices of regression analysis can lead to questionable results. This work highlights the application of simple *t*-tests for the detection of error due to measurement variability and presents a means to directly assess model error via flux profile simulation. At the same time, we bring attention to the impact of measurement variability on model identifiability, underlining the need for better reporting. Although this work has focused on the validation of a traditional MFA model via *t*-test analysis, the overall framework is likely to be just as applicable to other regression validation methods or alternative MFA formulations (such as dynamic MFA).

## Endnotes

^1^ A more detailed discussion of the theoretical principles, including a worked example and some proofs, is available as an Additional file 1.

^2^ It is typically assumed that *S*_*c*_ is sufficient for the estimation of all *v*_*c*_ values. However, failure to observe a key metabolite may result in a case where not all values of *v*_*c*_ can be estimated despite *S*_*c*_ appearing determined or overdetermined. See [30] for details on stoichiometry matrix classification.

## Additional files

Additional file 1 Extended theoretical principles. To make the proposed protocol as accessible as possible, the theoretical section has been extended with a number extra details as well as a simplified example model. (PDF 229 kb)

Additional file 2 Detailed model description and metabolic map. Definitions of all reactions included in the metabolic model. (PDF 125 kb)

Additional file 3 SBML file. An SBML representation of the metabolic model. (XML 89 kb)

## Abbreviations

ATP: Adenosine triphosphate
CHO: Chinese hamster ovary
COBRA: Constraint-based reconstruction and analysis
FADH: Flavin adenine dinucleotide
GLS: Generalized least squares
MFA: Metabolic flux analysis
NADH: Nicotinamide adenine dinucleotide
NADPH: Nicotinamide adenine dinucleotide phosphate
NMR: Nuclear magnetic resonance
SBML: Systems biology markup language
TCA: Tricarboxylic acid [cycle]
