# Supplementary material for: Identifying model error in metabolic flux analysis – a generalized least squares approach
Source: BMC Syst Biol. 2016 Sep 13;10(1):91. doi: 10.1186/s12918-016-0335-7 (PMC5020535; doi:10.1186/s12918-016-0335-7)
Supplement: Additional file 1 — Extended theoretical principles. To make the proposed protocol as accessible as possible, the theoretical section has been extended with a number extra details as well as a simplified example model. (PDF 229 kb) [file 12918_2016_335_MOESM1_ESM.pdf]

METHODOLOGY

Identifying model error in metabolic flux analysis – A generalized least squares approach

Stanislav Sokolenko, Marco Quattrociocchi and Marc G Aucoin*

*Correspondence:
maucoin@uwaterloo.ca
Department of Chemical Engineering, University of Waterloo, 200 University Avenue West, N2L 3G1, Waterloo, ON, Canada
Full list of author information is available at the end of the article

Additional file 1 — Extended theoretical principles

To make the proposed protocol as accessible as possible, the theoretical section has been extended with a number extra details as well as a simplified example model.

Basic principles of MFA[1]

The basis of MFA stems from a mass balance on each intracellular compound (Equation 1).

$$\begin{aligned}\text{rate of change} = \; & \text{transport in} \; - \; \text{transport out} \\ & + \; \text{generation} \; - \; \text{consumption}\end{aligned} \tag{1}$$

The right hand side of the equation can be separated into a net rate of formation and a dilution term that results from increasing cellular volume through cell division (Equation 8),

$$\frac{\mathrm{d}C}{\mathrm{d}t} = r - \mu C \tag{2}$$

where $\frac{\mathrm{d}C}{\mathrm{d}t}$ is a column vector of metabolite rates of change, r is a column vector of net fluxes, and μC is the dilution term. r combines transport fluxes and consumption/production from reactions (which include biomass production). Due to high turnover rate of intracellular metabolite pools, both $\frac{\mathrm{d}C}{\mathrm{d}t}$ and μC are generally assumed to be negligible in relation to large flux magnitudes (termed "pseudo-steady state"). The result is that the sum of transport and reaction fluxes must be equal to zero.

$$0 = r \tag{3}$$

The sum of transport and reaction fluxes can be expressed as a product of a stoichiometry matrix S and a column vector of fluxes v (Equation 4).

$$r = Sv \tag{4}$$

Each row of S corresponds to a metabolite, with the columns encoding the stoichiometry of reactions included in the model. As an example, a simple set of reactions is presented in Box 1, with the corresponding stoichiometry matrix defined in

[1]Mathematical description of MFA is largely based on [1].

Equation 5. Fluxes O1-O3 can be observed, while fluxes C1-C3 need to be calculated.

O1	$A_{external} \longrightarrow A$
O2	$B_{external} \longrightarrow B$
C1	$A + B \longrightarrow C$
C2	$C \longrightarrow D$
C3	$A + D \longrightarrow E$
O3	$E \longrightarrow E_{external}$

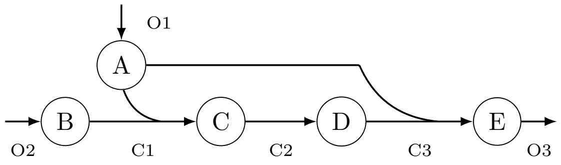

Box 1: Simple set of reactions defining a metabolic model.

$$S = \begin{array}{c} \\ A \\ B \\ C \\ D \\ E \end{array} \begin{array}{c} \begin{array}{cccccc} C1 & C2 & C3 & O1 & O2 & O3 \end{array} \\ \begin{bmatrix} -1 & 0 & -1 & 1 & 0 & 0 \\ -1 & 0 & 0 & 0 & 1 & 0 \\ 1 & -1 & 0 & 0 & 0 & 0 \\ 0 & 1 & -1 & 0 & 0 & 0 \\ 0 & 0 & 1 & 0 & 0 & -1 \end{bmatrix} \end{array} \tag{5}$$

Letters A, B, C, D, and E designate row names and correspond to balances on metabolites A, B, C, D, and E. C1, C2, C3, O1, O2, and O3 designate column names and refer to observed and calculated reactions. S can be partitioned into observed S_o and unknown (or calculated S_c) components,

$$Sv = S_c v_c + S_o v_o \tag{6}$$

where the observed fluxes tend to be metabolite uptake or secretion rates. For the reactions in Box 1,

$$S_o = \begin{array}{c} \\ A \\ B \\ C \\ D \\ E \end{array} \begin{array}{c} \begin{array}{ccc} O1 & O2 & O3 \end{array} \\ \begin{bmatrix} 1 & 0 & 0 \\ 0 & 1 & 0 \\ 0 & 0 & 0 \\ 0 & 0 & 0 \\ 0 & 0 & 1 \end{bmatrix} \end{array} \quad S_c = \begin{array}{c} \\ A \\ B \\ C \\ D \\ E \end{array} \begin{array}{c} \begin{array}{ccc} C1 & C2 & C3 \end{array} \\ \begin{bmatrix} -1 & 0 & -1 \\ -1 & 0 & 0 \\ 1 & -1 & 0 \\ 0 & 1 & -1 \\ 0 & 0 & -1 \end{bmatrix} \end{array} \tag{7}$$

Combining Equations (3), (4), and (6),

$$0 = S_c v_c + S_o v_o \tag{8}$$

Since S_o, S_c and v_o are all known, v_c can then be calculated. Going back to the example, assume that the uptakes of A and B have been observed as 2.1 and 0.9, while the secretion of E was found to be 1.2:

$$0 = S_c v_c + S_o v_o \tag{9}$$

$$0 = \begin{array}{c} \\ A \\ B \\ C \\ D \\ E \end{array} \begin{array}{c} \begin{array}{ccc} C1 & C2 & C3 \end{array} \\ \begin{bmatrix} -1 & 0 & -1 \\ -1 & 0 & 0 \\ 1 & -1 & 0 \\ 0 & 1 & -1 \\ 0 & 0 & 1 \end{bmatrix} \end{array} \begin{array}{c} C1 \\ C2 \\ C3 \end{array} \begin{bmatrix} v_{C1} \\ v_{C2} \\ v_{C3} \end{bmatrix} + \begin{array}{c} \\ A \\ B \\ C \\ D \\ E \end{array} \begin{array}{c} \begin{array}{ccc} O1 & O2 & O3 \end{array} \\ \begin{bmatrix} 1 & 0 & 0 \\ 0 & 1 & 0 \\ 0 & 0 & 0 \\ 0 & 0 & 0 \\ 0 & 0 & -1 \end{bmatrix} \end{array} \begin{array}{c} O1 \\ O2 \\ O3 \end{array} \begin{bmatrix} 2.1 \\ 0.9 \\ 1.2 \end{bmatrix} \tag{10}$$

The calculation and validation of v_c can be approached in multiple ways.

Traditional calculation

Assuming that sufficient fluxes can be observed, Equation (8) can be used to solve for the value of v_c through linear algebra:

$$0 = S_c v_c + S_o v_o \tag{11}$$
$$-S_c v_c = S_o v_o \tag{12}$$

To isolate for v_c, S_c must be invertible. If S_c has more rows than columns (and all rows are independent), then multiply both sides by S_c^T. Since $S_c^T S_c$ is square, it's possible to calculate v_c using a matrix inverse.

$$(S_c^T) - S_c v_c = (S_c^T) S_o v_o \tag{13}$$
$$v_c = -\left(S_c^T S_c\right)^{-1} S_c^T S_o v_o \tag{14}$$

For the example model:

$$\begin{matrix} C1 \\ C2 \\ C3 \end{matrix} \begin{bmatrix} 0.975 \\ 1.050 \\ 1.125 \end{bmatrix} \tag{15}$$

If v_o is known exactly, then Equation (14) can be substituted back into Equation (8) to define a redundency matrix R, which represents the flux balances around each metabolite as a function of the observed fluxes alone.

$$0 = S_c \left[-\left(S_c^T S_c\right)^{-1} S_c^T S_o v_o\right] + S_o v_o \tag{16}$$
$$0 = \left[-S_c \left(S_c^T S_c\right)^{-1} S_c^T S_o\right] v_o + S_o v_o \tag{17}$$
$$0 = \left[S_o - S_c \left(S_c^T S_c\right)^{-1} S_c^T S_o\right] v_o \tag{18}$$
$$0 = R v_o \tag{19}$$

For the example model:

$$R = \begin{matrix} \\ A \\ B \\ C \\ D \\ E \end{matrix} \begin{matrix} O1 & O2 & O3 \\ \left[\begin{matrix} 0.3333 & -0.3333 & -0.3333 \\ -0.3333 & 0.5833 & 0.0833 \\ 0 & 0.2500 & -0.2500 \\ 0 & 0.2500 & -0.2500 \\ 0.3333 & -0.0833 & -0.5833 \end{matrix}\right] \end{matrix} \tag{20}$$

Given that $R v_o = 0$, each row of R represents a relationship that must be satsfied by v_{O1}, v_{O2}, and v_{O3}. For example the balance on A will only hold if $v_{O1} = v_{O2} + v_{O3}$. Whereas v_o is a set of ideal observations that satisfy the balance perfectly, fluxes observed in practice ($\hat{v}_o$) are subject to experimental error and will result in some of the flux balances failing to close:

$$\varepsilon = R\hat{v}_o \tag{21}$$

where ε is the vector of residual fluxes associated with the flux balances around each of the metabolites (the length of ε is equal to the number of metabolites in the model). For the example model:

$$\varepsilon = \begin{array}{c} \\ A \\ B \\ C \\ D \\ E \end{array} \begin{array}{c} \begin{array}{ccc} O1 & O2 & O3 \end{array} \\ \begin{bmatrix} 0.3333 & -0.3333 & -0.3333 \\ -0.3333 & 0.5833 & 0.0833 \\ 0 & 0.2500 & -0.2500 \\ 0 & 0.2500 & -0.2500 \\ 0.3333 & -0.0833 & -0.5833 \end{bmatrix} \end{array} \begin{array}{c} O1 \\ O2 \\ O3 \end{array} \begin{bmatrix} 2.1 \\ 0.9 \\ 1.2 \end{bmatrix} \tag{22}$$

$$= \begin{array}{c} A \\ B \\ C \\ D \\ E \end{array} \begin{bmatrix} 0 \\ -0.075 \\ -0.075 \\ -0.075 \\ -0.075 \end{bmatrix} \tag{23}$$

By considering $\hat{v}_o$ as the sum of hypothetically true values (v_o) and a vector of observation errors (δ),

$$\hat{v}_o = v_o + \delta \tag{24}$$

it's possible to describe ε as a function of observation error alone. Starting from Equation 21:

$$\begin{aligned} \varepsilon &= R\hat{v}_o \\ &= R(v_o + \delta) \end{aligned} \tag{25}$$

Given that $Rv_o = 0$ by definition:

$$\varepsilon = R\delta \tag{26}$$

Relationships among the reactions in the metabolic model will cause linear dependencies in the redundancy matrix (R). In the simple reaction network of $A \to B \to C$, for example, a discrepancy in the observed transport fluxes of A and C will cause the same residual fluxes around A, B, and C, stemming from three linearly dependent rows in R. The degree of freedom that can be used to assess whether a significant observation error has been made is equal to the number of independent rows (rank) of R. For this reason, the redundancy matrix is reduced to only the linearly independent rows (R'), resulting in a corresponding change to ε (ε'). For the example model, R has a rank of two, so R' can be taken as two linearly independent rows of R:

$$R' = \begin{array}{c} \begin{array}{ccc} O1 & O2 & O3 \end{array} \\ \begin{bmatrix} 0.3333 & -0.3333 & -0.3333 \\ 0 & 0.2500 & -0.2500 \end{bmatrix} \end{array} \tag{27}$$

The vector of residuals ε is similarly reduced to ε':

$$\varepsilon' = R'\hat{v}_o \tag{28}$$

$$\varepsilon' = R'\delta \tag{29}$$

For the example model:

$$\varepsilon' = \begin{array}{c} \\ \\ \end{array}\begin{matrix} O1 & O2 & O3 \\ \end{matrix} \begin{bmatrix} 0.3333 & -0.3333 & -0.3333 \\ 0 & 0.2500 & -0.2500 \end{bmatrix} \begin{matrix} O1 \\ O2 \\ O3 \end{matrix} \begin{bmatrix} 2.1 \\ 0.9 \\ 1.2 \end{bmatrix} \tag{30}$$

$$= \begin{bmatrix} 0 \\ -0.075 \end{bmatrix} \tag{31}$$

The test for "gross measurement error" consists of determining whether the residual fluxes (ε') are normally distributed. If this is the case, then it can be concluded that the residuals are due to random noise in the observations. If the residuals are not normally distributed, at least one of the observations must contain a significant (gross) error. The sum of squares of standard normally distributed ($\mathcal{Z}$) random variables (x_i) is known to be chi-squared (χ^2) distributed. Expressed mathematically:

$$x_i \sim \mathcal{Z} \tag{32}$$

$$x = [x_1, x_2, \cdots, x_{n-1}, x_n]^T \tag{33}$$

$$\sum_{i=1}^{n}(x_i^2) \sim \chi^2 \tag{34}$$

The sum of squares can also be expressed in linear algebra as:

$$\sum_{i=1}^{n}(x_i^2) = x^T x \tag{35}$$

$$x^T x \sim \chi^2 \tag{36}$$

From this relationship, the "gross measurement error" test is a χ^2 test on the sum of squares of (row-reduced) residual fluxes, with the ε' terms standard normal variance of 1. The χ^2 statistic, h, is estimated as follows:

$$h = \varepsilon'^T \mathrm{Cov}(\varepsilon')^{-1}\varepsilon' \tag{37}$$

$$\sim \chi^2 \tag{38}$$

$\mathrm{Cov}(\varepsilon')$ is the variance-covariance matrix of ε' can be calculated using (26):

$$\varepsilon' = R'\delta$$

$$\mathrm{Cov}(\varepsilon') = \mathrm{Cov}(R'\delta) \tag{39}$$

$$= R'\mathrm{Cov}(\delta)R'^T \tag{40}$$

The transition from Equation (39) to Equation (40) can be shown from the definition of a variance-covariance matrix:

$$\mathrm{Cov}(X) = \mathrm{E}([X - \mathrm{E}(X)][X - \mathrm{E}(X)]^T) \tag{41}$$

$$\text{Cov}(RX) = \text{E}([RX - \text{E}(RX)][RX - \text{E}(RX)]^T) \tag{42}$$

Since $\text{E}(RX) = R\text{E}(X)$,

$$\begin{aligned} \text{Cov}(RX) &= \text{E}([RX - R\text{E}(X)][RX - R\text{E}(X)]^T) &(43)\\ &= \text{E}([(R)(X - \text{E}(X))][(R)(X - \text{E}(X))]^T) &(44) \end{aligned}$$

Since $(RX)^T = X^T R^T$

$$\begin{aligned} \text{Cov}(RX) &= \text{E}(R[X - \text{E}(X)][X - \text{E}(X)]^T R^T) &(45)\\ &= R\text{E}([X - \text{E}(X)][X - \text{E}(X)]^T)R^T &(46)\\ &= R\text{Cov}(X)R^T &(47) \end{aligned}$$

$\text{Cov}(\delta)$ is a covariance matrix of observed flux values that can be calculated directly through replication or estimated from prior information. For the example problem, v_o was taken to be $[2.1, 0.9, 1.2]^T$. Following the estimation of a covariance matrix around the observations, the χ^2 would be carried out as follows:

$$\text{Cov}(\delta) = \begin{array}{c} \\ O1 \\ O2 \\ O3 \end{array} \begin{array}{c} \begin{array}{ccc} O1 & O2 & O3 \end{array} \\ \begin{bmatrix} 0.01 & 0 & 0 \\ 0 & 0.01 & 0 \\ 0 & 0 & 0.04 \end{bmatrix} \end{array} \tag{48}$$

$$\text{Cov}(\varepsilon') = \begin{array}{c} \begin{array}{ccc} O1 & O2 & O3 \end{array} \\ \begin{bmatrix} 0.3333 & -0.3333 & -0.3333 \\ 0 & 0.2500 & -0.2500 \end{bmatrix} \end{array}$$

$$\cdot \begin{array}{c} \\ O1 \\ O2 \\ O3 \end{array} \begin{array}{c} \begin{array}{ccc} O1 & O2 & O3 \end{array} \\ \begin{bmatrix} 0.01 & 0 & 0 \\ 0 & 0.01 & 0 \\ 0 & 0 & 0.04 \end{bmatrix} \end{array} \begin{array}{c} O1 \\ O2 \\ O3 \end{array} \begin{bmatrix} 0.3333 & 0 \\ -0.3333 & 0.2500 \\ -0.3333 & -0.2500 \end{bmatrix} \tag{49}$$

$$= \begin{bmatrix} 0.006667 & 0.002500 \\ 0.002500 & 0.003125 \end{bmatrix} \tag{50}$$

$$h = \begin{bmatrix} 0 & -0.075 \end{bmatrix} \begin{bmatrix} 0.006667 & 0.002500 \\ 0.002500 & 0.003125 \end{bmatrix} \begin{bmatrix} 0 \\ -0.075 \end{bmatrix} \tag{51}$$

$$= 2.5714 \tag{52}$$

Since 2.5714 is smaller than the critical value of $\chi^2_{0.05} = 5.991$ for two degrees of freedom, no gross errors can be identified in the observations.

The suggested procedure is to use the χ^2 test to identify whether any significant errors are present among the observations. If no gross errors are identified then all observed values must contain only random noise, which can be "balanced" by finding an estimate for the observed values that minimizes the sum of squared errors for the observations ($\bar{v}_o$) given by:

$$\bar{v}_o = (I - \text{Cov}(\delta)R'^T \text{Cov}(\varepsilon')^{-1}R')\hat{v}_o \tag{53}$$

For the example problem, the corrected observations are $\bar{v}_o = [2.0571, 1.0286, 1.0286]^T$.

Generalized least squares calculation

Following the generalized least squares framework, the residual term ε is not assumed to be the result of measurement error alone and is introduced earlier in the formulation.

$$0 = S_c v_c + S_o v_o + \varepsilon \tag{54}$$

Since S_o and v_o are known, $S_o v_o$ is a column vector, and Equation (54) can be rearranged to look more like linear regression,

$$-S_o v_o = S_c v_c + \varepsilon \tag{55}$$

$$y = X\beta + \varepsilon \tag{56}$$

If the elements of ε are independently and identically distributed, then $\hat{v}_c$ can be estimated through ordinary regression (note that residuals don't have to be normally distributed for the estimate to be a best linear unbiased estimator).

$$\hat{\beta} = \left(X^T X\right)^{-1} X^T y \tag{57}$$

$$\hat{v}_c = -\left(S_c^T S_c\right)^{-1} S_c^T S_o v_o \tag{58}$$

Going back to the example model in Box 1,

$$S_o v_o = \begin{matrix} & O1 & O2 & O3 \\ A & 1 & 0 & 0 \\ B & 0 & 1 & 0 \\ C & 0 & 0 & 0 \\ D & 0 & 0 & 0 \\ E & 0 & 0 & -1 \end{matrix} \quad \begin{matrix} O1 \\ O2 \\ O3 \end{matrix} \begin{bmatrix} 2.1 \\ 0.9 \\ 1.2 \end{bmatrix} \tag{59}$$

$$= \begin{matrix} A \\ B \\ C \\ D \\ E \end{matrix} \begin{bmatrix} 2.1 \\ 0.9 \\ 0 \\ 0 \\ -1.2 \end{bmatrix} \tag{60}$$

$$\begin{matrix} A \\ B \\ C \\ D \\ E \end{matrix} \begin{bmatrix} 2.1 \\ 0.9 \\ 0 \\ 0 \\ -1.2 \end{bmatrix} = \begin{matrix} & C1 & C2 & C3 \\ A & -1 & 0 & -1 \\ B & -1 & 0 & 0 \\ C & 1 & -1 & 0 \\ D & 0 & 1 & -1 \\ E & 0 & 0 & 1 \end{matrix} \quad \begin{matrix} C1 \\ C2 \\ C3 \end{matrix} \begin{bmatrix} v_{C1} \\ v_{C2} \\ v_{C3} \end{bmatrix} \tag{61}$$

$$\hat{v}_c = \begin{matrix} C1 \\ C2 \\ C3 \end{matrix} \begin{bmatrix} 0.975 \\ 1.050 \\ 1.125 \end{bmatrix} \tag{62}$$

With this formulation, ε represents the deviation between observed and calculated fluxes that may be the result of either measurement error or lack of model fit. However, the assumption of ε being independently and identically distributed is unlikely to be true. Balances around large magnitude fluxes are likely to have higher associated variabilities. Mathematically, it is necessary to have $\mathrm{Cov}(\varepsilon) = \sigma^2$ with a variance-covariance matrix $\mathrm{Cov}(\varepsilon) = I\sigma^2$, whereas real balances may have unequal variances and non-zero covariance terms, equivalent to $\mathrm{Cov}(\varepsilon) = V\sigma^2$.

If Equation (55) is scaled by matrix A, then the variance-covariance of the scaled error term becomes

$$\mathrm{Cov}(A\varepsilon) = A\mathrm{Cov}(\varepsilon)A^T \tag{63}$$
$$= AV\sigma^2A^T \tag{64}$$
$$= \sigma^2AVA^T \tag{65}$$

where A must be chosen such that $AVA^T = I$ to meet the required conditions of linear regression. This is true if A is taken to be the inverse of the matrix square root of V, i.e., $A = P^{-1}$ and $PP = V$. Finding the square root of a matrix P, such that $PP = V$ can be performed by matrix diagonalization of V. To prove this, start from the matrix P and assume that that there exists a diagonal matrix D_P and matrix Γ, for which:

$$P = \Gamma D_P \Gamma^{-1} \tag{66}$$
$$PP = (\Gamma D_P \Gamma^{-1})(\Gamma D_P \Gamma^{-1}) \tag{67}$$
$$= \Gamma D_P D_P \Gamma^{-1} \tag{68}$$

Since $PP = V$ and the square of a diagonal matrix is that same matrix with all diagonal entries squared, then $D_P D_P$ can be redefined as diagonal matrix D_V:

$$V = \Gamma D_P D_P \Gamma^{-1} \tag{69}$$
$$V = \Gamma D_V \Gamma^{-1} \tag{70}$$

P can thus be calculated by diagonalizing V and taking the square root of its diagonal matrix, which is equal to the square root of the individual diagonal values. Since V is symmetric ($\mathrm{Cov}(X_1, X_2) = \mathrm{Cov}(X_2, X_1)$),

$$V = \Gamma D_V \Gamma^T \tag{71}$$

Therefore, P must also be symmetric:

$$P^T = (\Gamma D_P \Gamma^T)^T \tag{72}$$
$$= (\Gamma^T)^T (D_P)^T (\Gamma)^T \tag{73}$$
$$= \Gamma D_P \Gamma^T \tag{74}$$
$$= P \tag{75}$$

Combining $PP = V$ and $P = P^T$:

$$\mathrm{Cov}(P^{-1}\varepsilon) = P^{-1}\mathrm{Cov}(\varepsilon)(P^{-1})^T \tag{76}$$
$$= P^{-1}V\sigma^2(P^T) - 1 \tag{77}$$
$$= \sigma^2 P^{-1}PPP^{-1} \tag{78}$$
$$= \sigma^2 I \tag{79}$$

as required.

Scaling Equation (55) by P^{-1}:

$$-P^{-1}S_o v_o = P^{-1}S_c v_c + P^{-1}\varepsilon \tag{80}$$

where $P^{-1}\varepsilon$ now satisfies the assumptions of linear regression. Formally, this is equivalent to generalized least squares (GLS) regression, however, incorporating P^{-1} directly into each term allows the use of all ordinary least squares techniques:

$$-S'_o v_o = S'_c v_c + \varepsilon' \tag{81}$$

$$\hat{v}_c = -\left(S_c'^T S'_c\right)^{-1} S_c'^T S'_o v_o \tag{82}$$

The calculation of P^{-1} requires the estimation $\text{Cov}(\varepsilon)$ from the variance of observed fluxes. Calculating the covariance-variance matrix of both sides of Equation (55):

$$\text{Cov}(-S_o v_o) = \text{Cov}(S_c v_c + \varepsilon) \tag{83}$$

$$\text{Cov}(\varepsilon) = S_o \text{Cov}(v_o) S_o^T \tag{84}$$

Since $\text{Cov}(\varepsilon) = \sigma^2 V$ for any value of σ, σ is set to 1 so that $V = \text{Cov}(\varepsilon)$. In practice, $\text{Cov}(v_o)$ need only capture the relative magnitudes of observed flux variances as $\hat{\sigma}$ is estimated during regression. Balances around molecular species that do not include an observed flux v_o will have a row of zeros in $\text{Cov}(\varepsilon)$, which prevents the calculation of a matrix inverse (required to get P^{-1}). Although this mathematically equates to a variance of zero for those balance, a better interpretation is that there is an unknown variance around the "observation" of no net flux. The simplest solution is to add a small non-zero value to each diagonal entry of $\text{Cov}(\varepsilon)$, representing the confidence of the calculated fluxes being fully balanced. If there is more uncertainty around some balances than others, this information could be encoded in the magnitude of the added variance. P can then be calculated via a matrix square root of $\text{Cov}(\varepsilon)$. Since a variance (covariance) matrix is positive semi-definite, P is known to be unique.

Coming back to the example problem, assume that $\text{Cov}(v_o)$ has been defined as follows:

$$\text{Cov}(v_o) = \sigma^2 \begin{array}{c} \\ O1 \\ O2 \\ O3 \end{array} \begin{array}{c} \begin{array}{ccc} O1 & O2 & O3 \end{array} \\ \begin{bmatrix} 0.01 & 0 & 0 \\ 0 & 0.01 & 0 \\ 0 & 0 & 0.04 \end{bmatrix} \end{array} \tag{85}$$

Then,

$$\text{Cov}(\varepsilon) = \sigma^2 \begin{array}{c} \\ A \\ B \\ C \\ D \\ E \end{array} \begin{array}{c} \begin{array}{ccc} O1 & O2 & O3 \end{array} \\ \begin{bmatrix} 1 & 0 & 0 \\ 0 & 1 & 0 \\ 0 & 0 & 0 \\ 0 & 0 & 0 \\ 0 & 0 & -1 \end{bmatrix} \end{array}$$

$$\cdot \begin{array}{c} \\ O1 \\ O2 \\ O3 \end{array} \begin{array}{c} \begin{array}{ccc} O1 & O2 & O3 \end{array} \\ \begin{bmatrix} 0.01 & 0 & 0 \\ 0 & 0.01 & 0 \\ 0 & 0 & 0.04 \end{bmatrix} \end{array} \begin{array}{c} \\ O1 \\ O2 \\ O3 \end{array} \begin{array}{c} \begin{array}{ccccc} A & B & C & D & E \end{array} \\ \begin{bmatrix} 1 & 0 & 0 & 0 & 0 \\ 0 & 1 & 0 & 0 & 0 \\ 0 & 0 & 0 & 0 & -1 \end{bmatrix} \end{array} \tag{86}$$

$$= \sigma^2 \begin{array}{cc} & \begin{array}{ccccc} A & B & C & D & E \end{array} \\ \begin{array}{c} A \\ B \\ C \\ D \\ E \end{array} & \begin{bmatrix} 0.01 & 0 & 0 & 0 & 0 \\ 0 & 0.01 & 0 & 0 & 0 \\ 0 & 0 & 0 & 0 & 0 \\ 0 & 0 & 0 & 0 & 0 \\ 0 & 0 & 0 & 0 & 0.04 \end{bmatrix} \end{array} \tag{87}$$

Rows corresponding to balances on C and D do not include any observed fluxes, and are therefore all zero. The variance on the balance can be set to 0.0001 arbitrarily to reflect the relative confidence of the balance being closed:

$$\mathrm{Cov}(\varepsilon) = \sigma^2 V \tag{88}$$

$$\mathrm{Cov}(\varepsilon) = \sigma^2 \begin{array}{cc} & \begin{array}{ccccc} A & B & C & D & E \end{array} \\ \begin{array}{c} A \\ B \\ C \\ D \\ E \end{array} & \begin{bmatrix} 0.01 & 0 & 0 & 0 & 0 \\ 0 & 0.01 & 0 & 0 & 0 \\ 0 & 0 & 0.0001 & 0 & 0 \\ 0 & 0 & 0 & 0.0001 & 0 \\ 0 & 0 & 0 & 0 & 0.04 \end{bmatrix} \end{array} \tag{89}$$

Since V is a diagonal matrix, P^{-1} can be calculated as the reciprocal square root of each element.

$$P^{-1} = \begin{array}{cc} & \begin{array}{ccccc} A & B & C & D & E \end{array} \\ \begin{array}{c} A \\ B \\ C \\ D \\ E \end{array} & \begin{bmatrix} 10 & 0 & 0 & 0 & 0 \\ 0 & 10 & 0 & 0 & 0 \\ 0 & 0 & 100 & 0 & 0 \\ 0 & 0 & 0 & 100 & 0 \\ 0 & 0 & 0 & 0 & 5 \end{bmatrix} \end{array} \tag{90}$$

$$S'_o = \begin{array}{cc} & \begin{array}{ccccc} A & B & C & D & E \end{array} \\ \begin{array}{c} A \\ B \\ C \\ D \\ E \end{array} & \begin{bmatrix} 10 & 0 & 0 & 0 & 0 \\ 0 & 10 & 0 & 0 & 0 \\ 0 & 0 & 100 & 0 & 0 \\ 0 & 0 & 0 & 100 & 0 \\ 0 & 0 & 0 & 0 & 5 \end{bmatrix} \end{array} \begin{array}{cc} & \begin{array}{ccc} O1 & O2 & O3 \end{array} \\ \begin{array}{c} A \\ B \\ C \\ D \\ E \end{array} & \begin{bmatrix} 1 & 0 & 0 \\ 0 & 1 & 0 \\ 0 & 0 & 0 \\ 0 & 0 & 0 \\ 0 & 0 & -1 \end{bmatrix} \end{array} \tag{91}$$

$$= \begin{array}{cc} & \begin{array}{ccc} O1 & O2 & O3 \end{array} \\ \begin{array}{c} A \\ B \\ C \\ D \\ E \end{array} & \begin{bmatrix} 10 & 0 & 0 \\ 0 & 10 & 0 \\ 0 & 0 & 0 \\ 0 & 0 & 0 \\ 0 & 0 & -5 \end{bmatrix} \end{array} \tag{92}$$

$$S'_c = \begin{array}{cc} & \begin{array}{ccccc} A & B & C & D & E \end{array} \\ \begin{array}{c} A \\ B \\ C \\ D \\ E \end{array} & \begin{bmatrix} 10 & 0 & 0 & 0 & 0 \\ 0 & 10 & 0 & 0 & 0 \\ 0 & 0 & 100 & 0 & 0 \\ 0 & 0 & 0 & 100 & 0 \\ 0 & 0 & 0 & 0 & 5 \end{bmatrix} \end{array} \begin{array}{cc} & \begin{array}{ccc} C1 & C2 & C3 \end{array} \\ \begin{array}{c} A \\ B \\ C \\ D \\ E \end{array} & \begin{bmatrix} -1 & 0 & -1 \\ -1 & 0 & 0 \\ 1 & -1 & 0 \\ 0 & 1 & -1 \\ 0 & 0 & 1 \end{bmatrix} \end{array} \tag{93}$$

$$= \begin{array}{cc} & \begin{array}{ccc} C1 & C2 & C3 \end{array} \\ \begin{array}{c} A \\ B \\ C \\ D \\ E \end{array} & \begin{bmatrix} -10 & 0 & -10 \\ -10 & 0 & 0 \\ 100 & -100 & 0 \\ 0 & 100 & -100 \\ 0 & 0 & 5 \end{bmatrix} \end{array} \tag{94}$$

Performing the least squares calculation as before:

$$S'_o v_o = \begin{array}{cc} & \begin{array}{ccc} O1 & O2 & O3 \end{array} \\ \begin{array}{c} A \\ B \\ C \\ D \\ E \end{array} & \begin{bmatrix} 10 & 0 & 0 \\ 0 & 10 & 0 \\ 0 & 0 & 0 \\ 0 & 0 & 0 \\ 0 & 0 & -5 \end{bmatrix} \end{array} \begin{array}{c} O1 \\ O2 \\ O3 \end{array} \begin{bmatrix} 2.1 \\ 0.9 \\ 1.2 \end{bmatrix} \tag{95}$$

$$= \begin{matrix} A \\ B \\ C \\ D \\ E \end{matrix} \begin{bmatrix} 21 \\ 9 \\ 0 \\ 0 \\ -6 \end{bmatrix} \tag{96}$$

$$\begin{matrix} A \\ B \\ C \\ D \\ E \end{matrix} \begin{bmatrix} 21 \\ 9 \\ 0 \\ 0 \\ -6 \end{bmatrix} = \begin{matrix} \\ A \\ B \\ C \\ D \\ E \end{matrix} \begin{matrix} C1 & C2 & C3 \\ \begin{bmatrix} -10 & 0 & -10 \\ -10 & 0 & 0 \\ 100 & -100 & 0 \\ 0 & 100 & -100 \\ 0 & 0 & 5 \end{bmatrix} \end{matrix} \begin{matrix} C1 \\ C2 \\ C3 \end{matrix} \begin{bmatrix} v_{C1} \\ v_{C2} \\ v_{C3} \end{bmatrix} \tag{97}$$

$$\hat{v}_c = \begin{matrix} C1 \\ C2 \\ C3 \end{matrix} \begin{bmatrix} 1.0278 \\ 1.0287 \\ 1.0295 \end{bmatrix} \tag{98}$$

Whereas calculated fluxes $\hat{v}_c$ are commonly estimated using a very similar "weighted" least squares approach, the use of validation methods that are part of the regression framework have yet to be explored. The common χ^2 test can still be used to detect gross measurement errors in estimated residuals ($\hat{\varepsilon}$), however, the validation of a regression model also requires the use of t-tests to ensure the significance of calculated fluxes. Confidence and prediction intervals are also highly relevant to MFA. The calculation of a t-statistic follows from normal regression:

$$t_{\hat{\beta}_i} = \frac{\hat{\beta}_i}{\text{se}(\hat{\beta}_i)} \tag{99}$$

$$t_{\hat{v}_{c,i}} = \frac{\hat{v}_{c,i}}{\text{se}(\hat{v}_{c,i})} \tag{100}$$

$$t_{\hat{v}_{c,i}} = \frac{\left(-\left(S_c'^T S_c'\right)^{-1} S_c'^T S_o' v_o\right)_i}{\hat{\sigma}\sqrt{(S_c'^T S_c')^{-1}_{i,i}}} \tag{101}$$

The estimated standard deviation of ε (or $\hat{\sigma}$) is calculated as follows:

$$\hat{\sigma}^2 = \frac{\sum(\hat{\varepsilon}_i')^2}{n_b - n_c - 1} \tag{102}$$

where:

$$\hat{\varepsilon}' = -S_o' v_o + S_c' \left(S_c'^T S_c'\right)^{-1} S_c'^T S_o' v_o \tag{103}$$

and n_b is the number of balances (rows of S_c') while n_c is the number of fluxes to be calculated (columns of S_c'). If the model is correct and $\text{Cov}(\varepsilon)$ was correctly estimated, $\hat{\sigma}^2$ should be approximately equal to 1. Once the t-value is calculated, a flux can be judged statistically significant if $|t_{\hat{v}_{c,i}}| \geq t_{\alpha/2, n_b - n_c - 1}$ where α is the significance level.

As $\hat{v}_c$ has already been calculated for the example model, only $\hat{\sigma}$ and $\sqrt{(S_c'^T S_c')^{-1}_{i,i}}$ are required to perform a t-test.

$$\hat{\varepsilon}' = \begin{matrix} A \\ B \\ C \\ D \\ E \end{matrix} \begin{bmatrix} -0.4261 \\ 1.2784 \\ 0.0852 \\ 0.0852 \\ 0.8523 \end{bmatrix} \tag{104}$$

$$\hat{\sigma} = 1.60 \tag{105}$$

$$\sqrt{(S_c'^T S_c')^{-1}_{i,i}} = \begin{matrix} C1 \\ C1 \\ C3 \end{matrix} \begin{bmatrix} 0.0441 \\ 0.0442 \\ 0.0444 \end{bmatrix} \tag{106}$$

$$t_{0,\hat{v}_{c,i}} = \begin{matrix} C1 \\ C1 \\ C3 \end{matrix} \begin{bmatrix} 14.59 \\ 14.55 \\ 14.51 \end{bmatrix} \tag{107}$$

The critical t-value for a two-tailed test with 1 degree of freedom and 5% significance level is 12.71, making all three calculated fluxes statistically significant.

The identification of non-significant flux may be interpreted in two ways. The measurement error around observed fluxes may be too high to allow robust flux calculation. In that case, non-significant fluxes should be treated as having a flux of zero and excluded from the model or further analysis. Alternatively, non-significance may be the result of excess variability from a lack of fit between the model and observed data, requiring model correction. To distinguish between these cases, it is necessary to separate model error from measurement uncertainty. One way to accomplish this is to reduce measurement uncertainty through added replication, however, the required effort can make this approach practically infeasible. Another solution is to simulate a set of feasible fluxes directly from the stoichiometric model (and therefore free of model error) for comparison to the observed data.

The simulation of feasible fluxes can be simplified by eliminating flux equality constraints expressed by the stoichiometry matrix. Essentially, only $n_c - n_b$ fluxes have to specified in order to generate all the other values. More formally, the relationships between the fluxes can be succinctly summarized through the nullspace (or kernel) of S, which describes all flux balance conservations in the model. This makes it possible to calculate all fluxes from a smaller set of variables referred to as the basis. Unlike fluxes, which must satisfy constraints imposed by $Sv = 0$, the basis can take any arbitrary value to generate fluxes that satisfy all required constraints. Expressed mathematically,

$$\text{Null}(S) = K \tag{108}$$

$$Kb = v \tag{109}$$

where b is a basis vector of any value with the same number of rows as columns of K. While all values of b satisfy $Sv = 0$, it is still necessary to constrain fluxes to a set of realistic values representative of a cell cultivation. The space of all feasible fluxes v can be constrained by defining upper and lower bounds on each observed

flux:

$$\begin{aligned} & v = Kb \\ \text{subject to} \quad & K_i b \le v_i + a \cdot \text{sd}(v_i) \\ & K_i b \ge v_i - a \cdot \text{sd}(v_i) \end{aligned} \tag{110}$$

where v_i is an observed flux, K_i is the corresponding row of K, and a is a scaling constant that can be set to $t_{\alpha/2,df}$ to specify a confidence interval around v_i. As the basis solution space is only constrained by inequalities, it is readily amenable to stochastic sampling. All values of v that satisfy Equation (110) represent feasible fluxes that would perfectly satisfy the stoichiometric model while remaining within measurement uncertainty of real observations. If the resulting space is infeasible, then the observed data does not fit the specified model. Otherwise, a random sample of feasible fluxes can be taken for comparison to observed results. If the addition of measurement error to simulated fluxes results in less uncertainty than from observed results, then model error is to blame.

The example model can be solved by specifying only 1 flux value, meaning that K is a column vector.

$$K = \begin{matrix} C1 \\ C1 \\ C3 \\ O1 \\ O2 \\ O3 \end{matrix} \begin{bmatrix} 1 \\ 1 \\ 1 \\ 2 \\ 1 \\ 1 \end{bmatrix} \tag{111}$$

Based on K, a flux profile is consistent with the model as long as the flux of compound A (v_{O1}) is two times greater than all other fluxes. From the estimated variance-covariance matrix, the standard deviations of the observed fluxes are $[0.1, 0.1, 0.2]^T$. Assuming that these estimates came from a large number of samples, approximate 95% confidence intervals can be constructed by multiplying the standard deviation values by 2.

$$v = \begin{matrix} C1 \\ C1 \\ C3 \\ O1 \\ O2 \\ O3 \end{matrix} \begin{bmatrix} 1 \\ 1 \\ 1 \\ 2 \\ 1 \\ 1 \end{bmatrix} b \tag{112}$$

$$\begin{matrix} O1 \\ O2 \\ O3 \end{matrix} \begin{bmatrix} 2 \\ 1 \\ 1 \end{bmatrix} b \le \begin{matrix} O1 \\ O2 \\ O3 \end{matrix} \begin{bmatrix} 2.1 \\ 0.9 \\ 1.2 \end{bmatrix} + 2 \left(\begin{matrix} O1 \\ O2 \\ O3 \end{matrix} \begin{bmatrix} 0.1 \\ 0.1 \\ 0.2 \end{bmatrix} \right) \tag{113}$$

$$\begin{matrix} O1 \\ O2 \\ O3 \end{matrix} \begin{bmatrix} 2 \\ 1 \\ 1 \end{bmatrix} b \le \begin{matrix} O1 \\ O2 \\ O3 \end{matrix} \begin{bmatrix} 2.3 \\ 1.1 \\ 1.6 \end{bmatrix} \tag{114}$$

$$\begin{matrix} O1 \\ O2 \\ O3 \end{matrix} \begin{bmatrix} 2 \\ 1 \\ 1 \end{bmatrix} b \ge \begin{matrix} O1 \\ O2 \\ O3 \end{matrix} \begin{bmatrix} 2.1 \\ 0.9 \\ 1.2 \end{bmatrix} - 2 \left(\begin{matrix} O1 \\ O2 \\ O3 \end{matrix} \begin{bmatrix} 0.1 \\ 0.1 \\ 0.2 \end{bmatrix} \right) \tag{115}$$

$$\begin{matrix} O1 \\ O2 \\ O3 \end{matrix} \begin{bmatrix} 2 \\ 1 \\ 1 \end{bmatrix} b \geq \begin{matrix} O1 \\ O2 \\ O3 \end{matrix} \begin{bmatrix} 1.9 \\ 0.7 \\ 0.8 \end{bmatrix} \tag{116}$$

With only a single basis value b, the constraints can be combined to limit b between 0.95 and 1.1. Random values can then be generated from a uniform distribution. For basis vectors with more than one value, independent limits on values of b can not be isolated and the solution space must be sampled directly. Once basis vectors are generated, flux profiles can be calculated from $Kb = v$ and subject to measurement error.

References

1. Stephanopoulos, G., Aristidou, A., Nielsen, J.: Metabolic Engineering: Principles and Methodologies. Academic Press, San Diego (1998)
