# Supplementary material for: Identifying model error in metabolic flux analysis – a generalized least squares approach
Source: BMC Syst Biol. 2016 Sep 13;10(1):91. doi: 10.1186/s12918-016-0335-7 (PMC5020535; doi:10.1186/s12918-016-0335-7)
Supplement: Additional file 2 — Detailed model description and metabolic map. Definitions of all reactions included in the metabolic model. (PDF 125 kb) [file 12918_2016_335_MOESM2_ESM.pdf]

METHODOLOGY

Identifying model error in metabolic flux analysis – A generalized least squares approach

Stanislav Sokolenko, Marco Quattrociocchi and Marc G Aucoin*

*Correspondence:
maucoin@uwaterloo.ca
Department of Chemical Engineering, University of Waterloo, 200 University Avenue West, N2L 3G1, Waterloo, ON, Canada
Full list of author information is available at the end of the article

Additional file 2 – Model definition

The model used in this work was largely taken from [1] with only minimal changes (listed in the Materials and Methods section of the manuscript). A full list of reactions is presented below with a rough outline of matetrial flow in figure 1.

Glycolysis and PPP

$$
\begin{array}{r|l}
\text{HK} & \text{Glc} + \text{ATP} \longrightarrow \text{ADP} + \text{G6P} \\
\text{FBA} & \text{G6P} + \text{ATP} \longrightarrow \text{ADP} + 2\,\text{DHAP} \\
\text{PK} & \text{DHAP} + 2\,\text{ADP} + \text{NAD} + \text{P} \longrightarrow 2\,\text{ATP} + \text{NADH} + \text{Pyr} + \text{H}_2\text{O} \\
\text{PPP} & \text{G6P} + 2\,\text{NADP} + \text{H}_2\text{O} \longrightarrow 2\,\text{NADPH} + \text{R5P} + \text{CO}_2
\end{array}
$$

HK hexokinase
FBA fructose-biphosphate aldolase
PK pyruvate kinase
PPP pentose phosphate pathway

Pyr and AcCoA

$$
\begin{array}{r|l}
\text{LDH} & \text{Pyr} + \text{NADH} \longrightarrow \text{Lac} + \text{NAD} \\
\text{ACAS} & \text{mAcCoA} + \longrightarrow \text{mCoA} + \text{AcOH} \\
\text{ME} & \text{Mal} + \text{NADP} \longrightarrow \text{Pyr} + \text{CO}_2 + \text{NADPH} \\
\text{PDH} & \text{mPyr} + \text{mCoA} + \text{NAD} \longrightarrow \text{mAcCoA} + \text{NADH} + \text{CO}_2
\end{array}
$$

LDH lactate dehydrogenase
ACAS acetyl-coa synthetase
ME NADP-malic enzyme
PDH pyruvate dehydrogenase

TCA cycle

$$
\begin{array}{r|l}
\text{CS} & \text{mAcCoA} + \text{mOxal} + \text{H}_2\text{O} \longrightarrow \text{mCit} + \text{mCoA} \\
 & \text{mCit} + \text{NAD} \longrightarrow \text{mAKG} + \text{NADH} + \text{CO}_2 \\
\text{AKGDH} & \text{mAKG} + \text{mCoA} + \text{NAD} \longrightarrow \text{mSucCoA} + \text{NADH} + \text{CO}_2 \\
\text{SCS} & \text{ADP} + \text{mSucCoA} + \text{P} \longrightarrow \text{ATP} + \text{mCoA} + \text{mSuc} \\
 & \text{mFAD} + \text{mSuc} + \text{H}_2\text{O} \longrightarrow \text{mFADH} + \text{mMal} \\
\text{mMalDH} & \text{mMal} + \text{NAD} \longrightarrow \text{mOxal} + \text{NADH} \\
\text{cMalDH} & \text{NADH} + \text{Oxal} \longrightarrow \text{Mal} + \text{NAD}
\end{array}
$$

CS citrate synthase
AKGDH alpha-ketoglutarate dehydrogenase
SCS succinyl-coa synthetase
mMalDH mitochondrial malate dehydrogenase
cMalDH cytosolic malate dehydrogenase

Glutaminolysis

ALT	$\mathrm{Glu + Pyr \longrightarrow AKG + Ala}$
GLDH	$\mathrm{mGlu + NAD + H_2O \longrightarrow Amm + mAKG + NADH}$
GS	$\mathrm{Glu + ATP + Amm \longrightarrow Gln + ADP + P}$
AST	$\mathrm{Glu + Oxal \longrightarrow Asp + AKG}$
	$\mathrm{Glu \longrightarrow Pgl}$

ALT alanine transaminase
GLDH glutamate dehydrogenase
GS glutamine synthetase
AST aspartate transaminase

Amino acid degradation

SDH	$\mathrm{Ser \longrightarrow Pyr + Amm}$
SHMT	$\mathrm{Ser + FH_4 \longrightarrow Gly + N_5N_{10}methyleneFH_4 + H_2O}$
GCS	$\mathrm{CO_2 + Amm + N_5N_{10}methyleneFH_4 + NADH \longrightarrow Gly + FH_4 + NAD}$
	$\mathrm{Thr + mCoA + NAD \longrightarrow Gly + mAcCoA + NADH}$
	$\mathrm{Cys + AKG \longrightarrow Glu + Pyr}$
	$\mathrm{His + FH_4 + NADPH + 2\,H_2O \longrightarrow Glu + N_5N_{10}methyleneFH_4 + NADP + 2\,Amm}$
	$\mathrm{Arg + AKG + NAD + 2\,H_2O \longrightarrow 2\,Glu + NADH}$
	$\mathrm{Pro + 2\,NAD + 2\,H_2O \longrightarrow Glu + 2\,NADH}$
ASNS	$\mathrm{Asn + H_2O \longrightarrow Amm + Asp}$
	$\mathrm{Ile + AKG + ATP + 2\,mCoA + mFAD + 2\,NAD + H_2O \longrightarrow mSucCoA + Glu + mAcCoA + ADP + P + mFADH + 2\,NADH}$
	$\mathrm{Leu + ATP + AKG + NAD + mCoA + mFAD + H_2O + mSucCoA \longrightarrow 2\,mAcCoA + mSuc + Glu + NADH + mFADH + ADP + P}$
	$\mathrm{Lys + AKG + 2\,mCoA + 0.5\,O_2 + 2\,H_2O + 2\,mFAD + 2\,NAD \longrightarrow Glu + 2\,mAcCoA + 2\,CO_2 + Amm + 2\,mFADH + 2\,NADH}$
	$\mathrm{Met + Ser + 3\,ATP + FH_4 + 2\,NAD + ATP + mCoA + 4\,H_2O \longrightarrow mSucCoA + Cys + Amm + 3\,ADP + 3\,P + N_5N_{10}methyleneFH_4 + 2\,NADH + ADP}$
PAH	$\mathrm{Phe + NADPH + O_2 \longrightarrow Tyr + NADP + H_2O}$
	$\mathrm{Trp + 2\,mCoA + 3\,O_2 + 4\,H_2O + NADPH + 3\,NAD + mFAD \longrightarrow 2\,mAcCoA + 4\,CO_2 + Ala + NADP + 3\,NADH + mFADH + Amm}$
	$\mathrm{Tyr + AKG + mCoA + mSucCoA + 2\,O_2 + 2\,H_2O \longrightarrow Glu + Mal + 2\,mAcCoA + mSuc + CO_2}$
	$\mathrm{Val + AKG + 2\,ATP + mCoA + mFAD + 3\,NAD + 4\,H_2O \longrightarrow mSucCoA + Glu + 2\,ADP + 2\,P + mFADH + 3\,NADH + CO_2}$

SDH serine dehydratase
SHMT serine hydroxymethyltransferase
GCS glycine cleavage system
ASNS asparagine synthetase
PAH phenylalanine hydroxylase

Macromolecules

$$
\begin{array}{r|l}
\text{Carb} & \mathrm{G6P} + 3.5\,\mathrm{ATP} \longrightarrow \mathrm{Carb} + \mathrm{ADP} \\
\text{OA} & 9\,\mathrm{mCit} + 9\,\mathrm{Mal} + 17\,\mathrm{ATP} + 17\,\mathrm{NADPH} + 9\,\mathrm{NADH} + \mathrm{O_2} \longrightarrow \\
 & \qquad \mathrm{OA} + 9\,\mathrm{mMal} + 9\,\mathrm{Oxal} + 17\,\mathrm{ADP} + 17\,\mathrm{P} + 7\,\mathrm{NADP} + 9\,\mathrm{NAD} + \mathrm{H_2O} \\
\text{DNA} & 1.9\,\mathrm{Gln} + 1.3\,\mathrm{Asp} + 7.5\,\mathrm{ATP} + 0.5\,\mathrm{Gly} + 1.3\,\mathrm{N_5N_{10}methyleneFH_4} + \\
 & \qquad 0.7\,\mathrm{NAD} + 0.3\,\mathrm{NADPH} + \mathrm{R5P} + 0.3\,\mathrm{NADH} + 0.5\,\mathrm{CO_2} + 3.1\,\mathrm{H_2O} \longrightarrow \\
 & \qquad 1.9\,\mathrm{Glu} + 0.8\,\mathrm{Mal} + 7.5\,\mathrm{ADP} + 7.5\,\mathrm{P} + 1.3\,\mathrm{FH_4} + \\
 & \qquad 0.7\,\mathrm{NADH} + 0.3\,\mathrm{NADP} + 0.3\,\mathrm{NAD} + \mathrm{DNA} \\
\text{RNA} & 2.091\,\mathrm{Gln} + 1.194\,\mathrm{Asp} + 7.487\,\mathrm{ATP} + 0.489\,\mathrm{Gly} + 0.978\,\mathrm{N_5N_{10}methyleneFH_4} + \\
 & \qquad 0.806\,\mathrm{NAD} + 0.978\,\mathrm{NADP} + \mathrm{R5P} + 0.194\,\mathrm{NADH} + \\
 & \qquad 0.489\,\mathrm{CO_2} + 4.59\,\mathrm{H_2O} + 0.097\,\mathrm{O_2} \longrightarrow \\
 & \qquad 2.091\,\mathrm{Glu} + 0.683\,\mathrm{Mal} + 7487\mathrm{ADP} + 7.487\,\mathrm{P} + 0.978\,\mathrm{FH_4} + \\
 & \qquad 0.806\,\mathrm{NADH} + 0.978\,\mathrm{NADPH} + 0.194\,\mathrm{NAD} + \mathrm{RNA} \\
\text{Prot} & 0.095\,\mathrm{Ala} + 0.048\,\mathrm{Asp} + 0.039\,\mathrm{Asn} + 0.063\,\mathrm{Arg} + 0.028\,\mathrm{Cys} + \\
 & \qquad 0.052\,\mathrm{Gln} + 0.064\,\mathrm{Glu} + 0.078\,\mathrm{Gly} + 0.022\,\mathrm{His} + 0.052\,\mathrm{Ile} + \\
 & \qquad 0.088\,\mathrm{Leu} + 0.089\,\mathrm{Lys} + 0.02\,\mathrm{Met} + 0.021\,\mathrm{Phe} + 0.028\,\mathrm{Pro} + \\
 & \qquad 0.057\,\mathrm{Ser} + 0.061\,\mathrm{Thr} + 0.006\,\mathrm{Trp} + 0.02\,\mathrm{Tyr} + 0.059\,\mathrm{Val} + 4\,\mathrm{ATP} + 4\,\mathrm{H_2O} \longrightarrow \\
 & \qquad \mathrm{Prot} + 4\,\mathrm{ADP} + 4\,\mathrm{P}
\end{array}
$$

OA oleic acid (lipid) synthesis

Misc.

$$
\begin{array}{r|l}
 & \mathrm{mMal} + \mathrm{AKG} \longrightarrow \mathrm{mAKG} + \mathrm{Mal} \\
 & \mathrm{mMal} + \mathrm{Cit} \longrightarrow \mathrm{mCit} + \mathrm{Mal} \\
\text{GLAST} & \mathrm{Glu} \longrightarrow \mathrm{mGlu} \\
\text{MPC} & \mathrm{Pyr} \longrightarrow \mathrm{mPyr} \\
 & \mathrm{FH_4} + \mathrm{FoOH} + \mathrm{ATP} + \mathrm{NADH} \longrightarrow \mathrm{ADP} + \mathrm{P} + \mathrm{NAD} + \mathrm{N_5N_{10}methyleneFH_4}
\end{array}
$$

GLAST glutamate aspartate transporter
MPC mitochondrial pyruvate carrier

Phosphorylation

$$
\begin{array}{|l}
3\,\mathrm{ADP} + \mathrm{NADH} + 0.5\,\mathrm{O_2} + 3\,\mathrm{P} \longrightarrow 3\,\mathrm{ATP} + \mathrm{NAD} + 4\,\mathrm{H_2O} \\
2\,\mathrm{ADP} + \mathrm{mFADH} + 0.5\,\mathrm{O_2} + 2\,\mathrm{P} \longrightarrow 2\,\mathrm{ATP} + \mathrm{mFAD} + 2\,\mathrm{H_2O}
\end{array}
$$

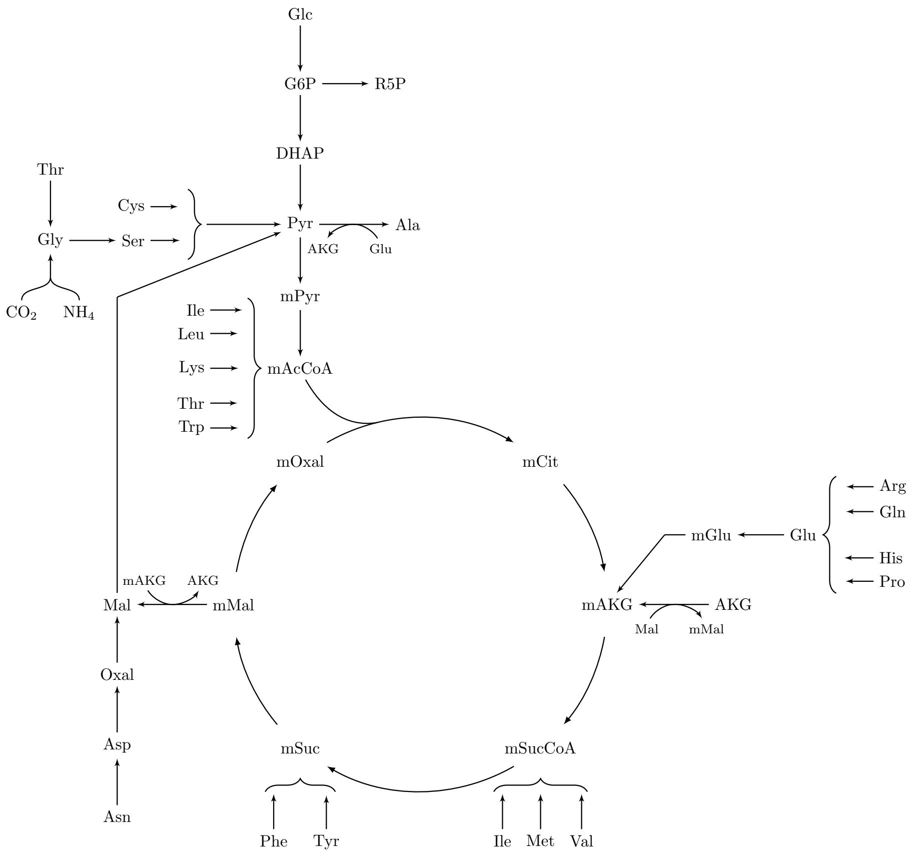

Figure 1 Outline of metabolic network used in this work. A number of intermediate compounds have been omitted to clarify overall material flow.

References

1. Altamirano, C., Illanes, A., Casablancas, A., Gámez, X., Cairó, J.J., Gòdia, C.: Analysis of CHO cells metabolic redistribution in a glutamate-based defined medium in continuous culture. Biotechnology Progress **17**(6), 1032–41 (2001). doi:10.1021/bp0100981
